# Source apportionment of eleven trace metals across six São Paulo river basins (1978–2026): receptor modelling identifies geogenic misregulation and a monitoring blind spot for diffuse-source zinc

**DOI:** 10.1007/s10653-026-03379-2

**Published:** 2026-08-18

**Authors:** Hugo Pimentel Tavares, Nilo Antônio de Souza Sampaio

**Affiliations:** Departamento de Engenharia Ambiental (DEAMB), Faculdade de Tecnologia (FAT), Universidade do Estado do Rio de Janeiro (UERJ), Resende, RJ 27537-000 Brazil

**Keywords:** Receptor modelling, Non-negative matrix factorisation, Trace metals, Geogenic contamination, Zinc, Tyre wear particles, São Paulo, CETESB, CONAMA, Environmental health, Long-term monitoring

## Abstract

Trace metal contamination of freshwater systems arises from a superposition of geogenic and anthropogenic sources that compliance monitoring cannot disentangle. This study applies Non-negative Matrix Factorisation (NMF) receptor modelling to 126,140 records of eleven trace metals (Al, As, Ba, Cd, Cu, Fe, Hg, Mn, Ni, Pb, Zn) at 114 monitoring stations across six water management units (UGRHIs) of São Paulo State, Brazil (1978–2026), operated by CETESB—one of the longest continuous total-recoverable multi-metal river monitoring records in Latin America. Four geochemically interpretable source factors are resolved: Fe–Al geogenic weathering (F1), Al soil erosion (F2), Fe–Mn redox/industrial effluents (F3), and Zn–Mn urban diffuse sources (F4). Mann–Kendall trend analysis (2000–2025) reveals two divergent trajectories: copper declines significantly (Cu, $\tau = -\,0.709$, p<0.001), consistent with CONAMA Resolution 357/2005 industrial-effluent enforcement, while the geogenic metals iron and aluminium also decline in a manner consistent with reduced erosional loading rather than effluent control, and median-based trends suggest rising urban-source metals (Zn, $\tau = +\,0.727$; Ni, $\tau = +\,0.461$). However, a censoring-invariant exceedance analysis introduced here demonstrates that these apparent increases are inflated by documented rises in analytical detection limits (Zn: 0.020–0.100 mg L$^{-1}$ in 2012): no metal exhibits a rising exceedance of its CONAMA Class 2 limit, and the fractions exceeding the limit for Zn and Ni in fact decline ($\tau = -\,0.477$ and -0.600, both p<0.001). System-wide aluminium (91.6%) and iron (88.2%) violation rates under CONAMA Class 2 are attributable primarily to geogenic Factors F1 and F2—tropical Latosol weathering—not to anthropogenic contamination, constituting a regulatory artefact with direct implications for environmental governance in high-weathering tropical geologies. Critically, the current Zn reporting limit (0.100 mg L$^{-1}$) sits above the chronic ecotoxicological threshold for sensitive freshwater species (10–$50~\upmu \text {g/L}$) and at 56% of the CONAMA limit, leaving the monitoring network structurally unable to resolve ecologically relevant Zn from tyre and road wear particles—a diffuse-source monitoring and governance gap absent from Brazil’s current regulatory architecture. These findings position São Paulo’s metal governance within the global pollution-transition pattern: measurable regulatory success for industrial point sources, alongside a detection-limit blind spot for emerging diffuse-source metals, with transferable methodological and policy implications for tropical regions where geogenic background geochemistry conflicts with standards calibrated to temperate-zone geology and where evolving detection limits can masquerade as concentration trends.

**Supplementary Information:**

The online version contains supplementary material available at https://doi.org/10.1007/s10653-026-03379-2.

## Introduction

The relationship between surface water geochemistry and human and ecosystem health is mediated by a mixture of natural (geogenic) and anthropogenic metal sources whose individual contributions compliance monitoring cannot resolve (Aura et al., 2024). Rivers in rapidly industrialising regions accumulate metal loads from heterogeneous inputs: industrial effluent, urban stormwater, agricultural runoff, atmospheric deposition, and the weathering of local lithologies—each demanding different governance responses, yet producing undifferentiated signals at a single monitoring station. This source-attribution gap is globally recognised: the US Environmental Protection Agency Positive Matrix Factorisation (EPA PMF) receptor modelling framework was developed explicitly to address it in atmospheric and aquatic systems (Paatero & Tapper, 1994); the European Water Framework Directive (WFD) requires member states to distinguish anthropogenic from natural background concentrations as a precondition for setting realistic quality standards; and a growing body of research has applied receptor modelling to rivers in Europe (Song et al., 2024), Asia (Li et al., 2022), and North America, but the technique remains essentially absent from the Brazilian freshwater literature.

São Paulo State, Brazil, hosts one of the most extensive long-term water quality monitoring networks in Latin America, operated by CETESB since 1978. Covering six major water management units (Unidades de Gerenciamento de Recursos Hídricos, UGRHIs), 114 monitoring stations, and 126,140 records of eleven trace metals, the CETESB database provides a data substrate unique in the Global South for receptor modelling applications: sufficient temporal depth (47 years) to resolve long-term trend superimposed on source-factor dynamics, and sufficient spatial density to map source-factor contributions across basins with contrasting land uses (agroindustrial, metallurgical, urbanised, and predominantly natural). Prior work by the present authors has characterised long-term CONAMA-compliance dynamics in Brazilian surface-water records—for conventional water-quality parameters in the neighbouring Paraíba do Sul basin (Pimentel Tavares et al., 2026), and for the latency with which routine monitoring detects sustained regulatory impairment in the present São Paulo network (Tavares & de Souza Sampaio, 2026); the present study extends that line of enquiry to the source apportionment of trace metals.

The regulatory context motivates the study. CONAMA Resolution 357/2005 (CONAMA, 2005) established Class 2 water quality standards for eleven trace metals studied here. Two striking anomalies in the compliance record demand explanation. First, aluminium and iron exhibit system-wide violation rates of 91.6% and 88.2% respectively—far exceeding any other metal—yet their concentrations show declining or stable trends, and they are not co-associated with classical industrial tracers such as Pb or Cd. This pattern is consistent with a geogenic origin: Brazilian Latosols and Ultisols, which dominate the São Paulo pedological landscape, contain aluminium and iron oxide fractions of 20–40% by mass, generating naturally elevated dissolved and colloidal loads in surface waters independent of any anthropogenic input (Zahan et al., 2023). The CONAMA limits, derived from WHO temperate-zone drinking water guidelines, do not account for this geological reality, potentially misdirecting enforcement resources toward non-remediable geogenic conditions while leaving genuinely anthropogenic sources less scrutinised. Second, zinc presents the strongest *apparent* increasing median trend of any metal in the dataset ($\tau = +\,0.727$, p<0.001). Zinc is not enriched in Brazilian Latosols and has no geogenic mechanism that would produce such a signal, and its urban diffuse attribution parallels the documented global phenomenon of zinc contamination from tyre and road wear particles (TRWP) (Baensch-Baltruschat et al., 2020), a source category absent from CONAMA’s current regulatory architecture. As we show, however, this median trend does not survive a censoring-invariant test and is largely an artefact of an evolving analytical reporting limit—a finding that, rather than dismissing zinc, redirects attention to a structural monitoring blind spot in the ecotoxicologically relevant concentration range. Disentangling this apparent trend from analytical reporting change is one of the central methodological contributions of the study.

Against this background, this study pursues four interlinked objectives: (i) to apply NMF receptor modelling to the CETESB multi-metal database and resolve statistically distinct source factors; (ii) to characterise the temporal trajectories of each resolved source factor using Mann–Kendall trend analysis; (iii) to map the spatial contribution of each source factor across the six UGRHIs and link the patterns to known land use and industrial activities; and (iv) to evaluate the regulatory implications of the geogenic source-factor findings, with specific reference to the misattribution of natural background Al and Fe loads as CONAMA violations, and to identify the emerging urban diffuse metal source (Factor 4, Zn–Mn) as the priority target for updated governance.

## Literature review

### Source apportionment of trace metals in river systems

Receptor modelling approaches, originally developed for atmospheric source attribution (Paatero & Tapper, 1994), have been increasingly applied to riverine trace metal datasets. The principal positive matrix factorisation (PMF) framework, implemented by the US EPA as EPA PMF 5.0, decomposes an observation matrix into source profiles (factor loadings) and station contributions (factor scores) without requiring a priori knowledge of source compositions, making it particularly valuable where direct source sampling is impractical or unavailable. The mathematically related Non-negative Matrix Factorisation (NMF) framework (Lee & Seung, 1999) applies the same non-negativity constraint—physically meaningful for concentration data, as negative source contributions are impossible—and has been demonstrated to produce source profiles consistent with PMF in comparative studies (Li et al., 2022).

Applications of receptor modelling to aqueous-phase metal fractions in river water (as opposed to sediments, which dominate the literature) remain comparatively scarce. Li et al. (2022) applied integrated source apportionment to long-term monitoring data (1999–2016) from typical surface water systems in China, identifying historical mining, industrial, agricultural, and geological natural sources as the main contributors and documenting temporal shifts in source mix following governance interventions—a methodological precedent closely analogous to the present study’s objectives. Source apportionment studies published in *Environmental Geochemistry and Health* have documented three to five-factor solutions for trace metals in sediments from Asian rivers and lakes (Cao et al., 2026; Song et al., 2024), with natural geogenic sources typically accounting for 25–50% of total metal variance in tropical and subtropical systems.

### Geogenic background and regulatory misattribution

The problem of regulatory misattribution—where water quality standards derived from temperate-zone background geochemistry flag geogenically elevated concentrations as violations in tropical high-weathering geologies—is globally documented but has received limited attention in the Brazilian context. In lateritic (Latosol/Ultisol) geologies, Al$_2$O$_3$ contents of 20–45% and Fe$_2$O$_3$ contents of 10–25% by mass produce dissolved and colloidal Al and Fe concentrations in surface waters that systematically exceed WHO-derived standards even in pristine catchments (Zahan et al., 2023). The US EPA has addressed analogous situations through the “reference condition” methodology (Clean Water Act Section 304(a)), which calibrates ambient water quality criteria to natural background concentrations; the WFD similarly requires member states to establish natural background concentrations as a baseline for metal quality standards. Brazil’s CONAMA 357/2005 lacks a geogenic correction mechanism, creating a structural regulatory artefact that receptor modelling can quantitatively characterise.

### Zinc, tyre wear particles, and urban diffuse sources

The global rise of zinc in urban freshwater systems has been linked to tyre and road wear particles (TRWP) as the primary emerging diffuse source (Baensch-Baltruschat et al., 2020). Tyres contain 1–4% zinc by mass, predominantly as zinc oxide (ZnO), incorporated as a vulcanisation activator and accelerant. Abrasion of tyres on roads generates TRWP at estimated rates of 100,000 t year$^{-1}$ in Germany alone (Baensch-Baltruschat et al., 2020), of which approximately 20,000 t are transported to surface waters via stormwater. In aquatic systems, zinc leaches rapidly from TRWP (90% release within 48 h at environmental pH) and has been documented as the primary metal driver of acute toxicity to coho salmon (*Oncorhynchus kisutch*) in US Pacific Northwest urban streams (Tian et al., 2021). The global vehicle fleet has grown from approximately 800 million (2000) to over 1.5 billion (2024) vehicles, with concentrated growth in rapidly urbanising regions including Brazil (14 million to 28 million vehicles in São Paulo State, 2000–2024). This parallel growth of vehicle fleets and Zn monitoring trends in rivers worldwide constitutes the global evidence framework within which the present study’s findings for São Paulo are positioned.

Nickel is a forward-looking, precautionary concern. NMC and NCA lithium-ion battery cathodes contain 40–80% nickel by mass; nickel-metal hydride batteries in hybrid vehicles contain 22–30%; and industrial nickel alloy infrastructure corrosion contributes diffuse loading to urban rivers (Aura et al., 2024). As we show, nickel’s apparent median increase is—like that of zinc—an artefact of its censoring structure rather than a genuine concentration rise; the value of the present record is therefore as a pre-transition baseline against which any future increase could be detected, should Brazil’s fleet electrification under the Mover programme (target: 30% electrified sales by 2030) generate one, provided the reporting limit is lowered into the ecotoxicologically relevant range.

### Long-term governance effectiveness in Brazilian river basins

The CETESB monitoring record is among the most extensive long-running water quality datasets in the Global South. Studies of comparable long-term networks worldwide—the Yangtze River sub-basins in China (Yang et al., 2023), mining-affected basins in the Quadrilátero Ferrífero in Minas Gerais (Silva et al., 2026), and urban river systems across Asia (Nguyen et al., 2026)—consistently report that regulatory milestones produce heterogeneous outcomes, with industrial point-source parameters responding more rapidly than diffuse agricultural or urban loads. The São Paulo network exhibits a similar structural pattern: compliance improvements in parameters targeted by CONAMA 357/2005 (e.g. dissolved oxygen in some basins) have occurred concurrently with deterioration in others (nutrients, certain trace metals). This governance paradox is the analytical starting point for the present receptor modelling study, which provides the metal contamination dimension of São Paulo’s multi-parameter impairment profile for the first time by decomposing observed metal loads into geogenic and anthropogenic source factors—an approach that aggregate compliance reporting cannot offer.

## Materials and methods

### Dataset and study area

The primary dataset comprises 646,356 physicochemical records from 141 CETESB monitoring stations distributed across São Paulo State, Brazil (CETESB QUALAR database, accessed January 2026). Of these, 126,140 records correspond to the eleven trace metals analysed: aluminium (Al), arsenic (As), barium (Ba), cadmium (Cd), copper (Cu), iron (Fe), mercury (Hg), manganese (Mn), nickel (Ni), lead (Pb), and zinc (Zn). The study area encompasses six water management units (UGRHIs): 02 – Paraíba do Sul (22 stations, 11 metals), 05 – Piracicaba/Capivari/Jundiaí (56 stations, 11 metals), 06 – Alto Tietê (10 stations, 11 metals), 09 – Mogi Guaçu (17 stations, 11 metals), 10 – Sorocaba/Médio Tietê (6 stations, 11 metals), and 13 – Tietê/Jacaré (3 stations, 10 metals). The study period spans 1978–2026, constituting one of the longest continuous total-recoverable multi-metal monitoring records in Latin America. All 114 stations with metal data include geographic coordinates (latitude, longitude), regulatory classification (CLASSE), and municipal attribution. Concentration values are stored with comma-decimal notation in the source database (e.g., 1,760000 for 1.76 mg L$^{-1}$) and were converted to numeric format prior to analysis.

The six UGRHIs present contrasting land use contexts (Table 1). UGRHI 05 (Piracicaba/Capivari/Jundiaí) is the most data-rich and is characterised by intensive sugarcane, citrus, and soybean monoculture combined with a large agroindustrial processing sector. UGRHI 06 (Alto Tietê) encompasses the São Paulo Metropolitan Region with a population of approximately 22 million. UGRHI 10 (Sorocaba/Médio Tietê) hosts a concentrated industrial corridor of approximately 3000 metalworking, automotive parts, paper/pulp, and textile establishments. UGRHI 09 (Mogi Guaçu) is predominantly rural with forest cover in the headwaters. These contrasting settings provide a natural experiment for differentiating geogenic from anthropogenic factor contributions across basins.

**Table 1:** UGRHI characteristics and metal monitoring coverage

UGRHI	Name	Stations	Metals	Records
02	Paraíba do Sul	22	11	22,741
05	Piracicaba/Capivari/Jundiaí	56	11	65,132
06	Alto Tietê	10	11	15,291
09	Mogi Guaçu	17	11	14,729
10	Sorocaba/Médio Tietê	6	11	6551
13	Tietê/Jacaré	3	10	1696
Total				126,140

UGRHI (Unidade de Gerenciamento de Recursos Hídricos): the administrative water management unit subdivision used for water resource planning and monitoring in São Paulo State, Brazil. Six of the 22 UGRHIs in São Paulo State are covered by this study

### Study area and monitoring network

**Fig. 1:**
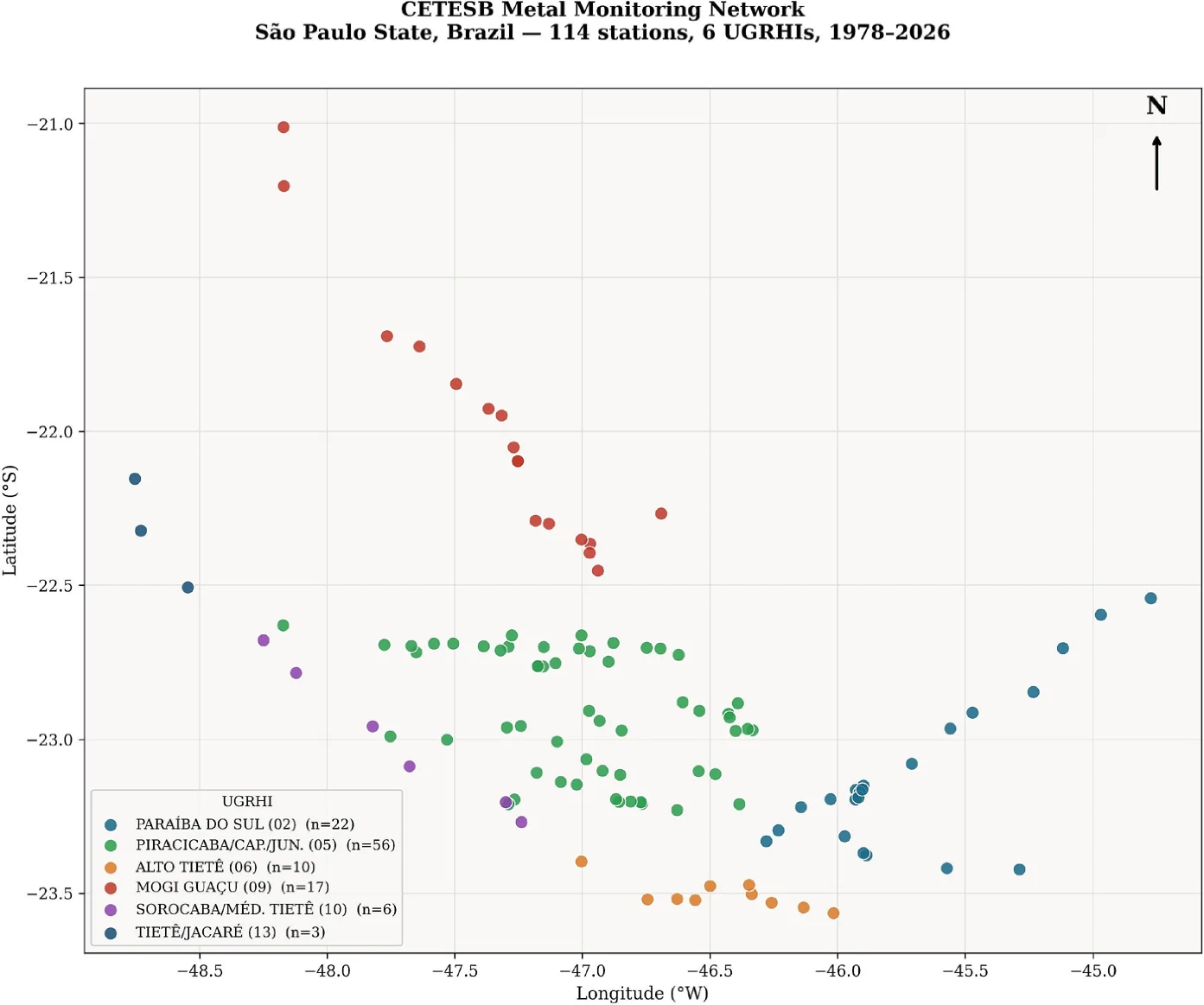
Spatial distribution of the 114 CETESB metal monitoring stations across six water management units (UGRHIs) in São Paulo State, Brazil. Coordinates retrieved directly from the CETESB QUALAR database (accessed January 2026) and converted from degrees–minutes–seconds to decimal degrees. Colour coding identifies UGRHI membership; station counts per UGRHI are given in Table 1

Figure 1 presents the spatial distribution of the 114 CETESB monitoring stations with metal data across the six UGRHIs. Stations span the full latitudinal ($-\,23.6^{\circ }$ to $-\,21.0^{\circ }$S) and longitudinal ($-\,48.8^{\circ }$ to $-\,44.8^{\circ }$W) extent of the study area, covering contrasting land uses from the metropolitan region of São Paulo (UGRHI 06) to predominantly rural headwaters (UGRHI 09). Geographic coordinates (in degrees–minutes–seconds) were retrieved directly from the CETESB QUALAR database and converted to decimal degrees for mapping.

### Analytical quality assurance of source data

This study uses secondary monitoring data from the CETESB QUALAR open-access database. As is standard for national-scale institutional monitoring networks (e.g., US Geological Survey NWIS; European Environment Agency WFD-WISE), instrument-level parameters such as specific instrumentation per metal, certified reference material recoveries, and individual batch blanks are not individually reported in the public portal. The following describes the institutional quality framework within which all reported data were produced.

The CETESB laboratory network holds accreditation from the Brazilian National Institute of Metrology, Quality and Technology (INMETRO/CGCRE—Coordenação Geral de Acreditação) under ABNT NBR ISO/IEC 17025 (CETESB, 2024), the international standard for laboratory testing competence. CETESB laboratories are members of the Rede Brasileira de Laboratórios de Ensaio (RBLE) and the Red de Laboratorios de Ambiente y Salud de América Latina y El Caribe (RELAC).

Field sampling across the network follows CETESB’s standardised surface-water protocol, harmonised with the Brazilian national guide for sample collection and preservation (ANA and CETESB, 2011): sub-surface grab samples are collected at fixed georeferenced stations and, for total metals, preserved by acidification (nitric acid to pH <2) and subjected to acid digestion prior to analysis, consistent with Standard Methods 3030 (APHA, 2017). Because the QUALAR portal distributes validated analytical results rather than field metadata, per-sample collection depth and container-preparation records are not individually retrievable, as is typical of secondary use of institutional monitoring data. We note, however, that the concern that routine (as opposed to trace-metal-clean) sampling might introduce contamination artefacts is material chiefly at the sub-microgram-per-litre concentrations encountered in pristine or open-water trace-metal research. The metals that drive the conclusions of this study occur at concentrations two to four orders of magnitude higher—median Fe and Al near 1–2 mg L$^{-1}$, and Zn, Cu and Mn at tens to hundreds of micrograms per litre—so that incidental contamination during grab sampling is negligible relative to the environmental signals analysed. The metals for which sampling contamination could in principle be non-trivial (Cd, Pb at low-microgram levels) are precisely those excluded from quantitative interpretation on censoring grounds (Table 2), so no conclusion rests on them.

Analytical methods follow the *Standard Methods for the Examination of Water and Wastewater* (APHA, 2017). The CETESB Inorganic Chemistry Laboratory publicly documents the instrumental techniques employed for environmental monitoring:

(i)  graphite furnace atomic absorption spectrometry (GF-AAS) is used for Al, As, Pb, and Cd, which require ultra-trace sensitivity below ICP-OES detection thresholds for the relevant regulatory concentrations;
(ii)  atomic fluorescence spectrometry (AFS) is used for Hg and methyl-Hg, providing sub-nanogram sensitivity consistent with the CONAMA Class 2 limit of 0.0002 mg L$^{-1}$;
(iii)  inductively coupled plasma optical emission spectrometry (ICP-OES) is used for multi-element determination of Fe, Mn, Cu, Zn, Ni, Ba, and related parameters;
(iv)  inductively coupled plasma mass spectrometry (ICP-MS) is used for trace-level multi-element determinations where lower detection limits are required (CETESB-Lab, 2025).

The parameter nomenclature in CETESB QUALAR designates all metals as *Total* (e.g., Ferro Total, Zinco Total), indicating total-recoverable determinations on unfiltered samples subjected to acid digestion prior to analysis, consistent with Standard Methods 3030 pre-treatment protocols for surface water matrices (APHA, 2017).

Within the CETESB QUALAR portal, measurements recorded below the method detection limit (MDL) are flagged with a less-than symbol ($\texttt {`<'}$) in the sign (*Sinal*) field. Table 3 reports the proportion of below-detection records and number of stations with imputed values per metal. Metals with high censoring rates—Hg (86.4%) and As (97.5%)—are analysed using Kaplan–Meier and maximum likelihood estimation for censored data (“Kaplan–Meier and maximum likelihood estimation for censored metals” section). All numerical values were extracted directly from the CETESB QUALAR portal (accessed January 2026) and verified against the raw database prior to analysis.

Table 2 reports, for each metal, the analytical technique, the range of reported detection limits across the trend-analysis period, the CONAMA Class 2 limit, and the overall below-detection fraction. It serves two purposes: it provides the per-metal detection-limit information required to interpret the compliance and trend results, and it documents the evolution of reporting limits that is central to the censoring-robust trend analysis of “Censoring-robust trend verification” section. Nine of the eleven metals experienced a change in reported MDL of a factor of two or more; aluminium alone retained a stable reporting limit (0.100 mg L$^{-1}$) and thus acts as an internal control confirming that concentration declines can be resolved despite the general instability of reporting limits.

**Table 2:** Analytical technique, reported method detection limit (MDL) range, CONAMA Class 2 limit, and below-detection fraction for each of the eleven trace metals over the full monitoring record (CETESB QUALAR, 1978–2026)

Metal	Technique	MDL range (mg L$^{-1}$)	CONAMA	<MDL (%)	MDL $\ge 2\times$ change
Al	GF-AAS	0.1	0.1	7	No
Fe	ICP-OES	0.1–0.3	0.3	3	Yes
Mn	ICP-OES	0.002–0.1	0.1	18	Yes
Cu	ICP-OES	0.005–0.01	0.009	56	Yes
Zn	ICP-OES	0.02–0.1	0.18	49	Yes
Ni	ICP-OES	0.02	0.025	72	No
Ba	ICP-OES	0.02–0.08	0.7	38	Yes
Cd	GF-AAS	0.0001–0.005	0.001	90	Yes
Pb	GF-AAS	0.01–0.1	0.01	81	Yes
As	GF-AAS	0.002–0.01	0.01	97	Yes
Hg	AFS	0.0001–0.0002	0.0002	86	Yes

The MDL range is the span of the modal reported detection limit across successive five-year windows within the period, documenting the evolution of analytical reporting practice that motivates the censoring-robust trend analysis (“Censoring-robust trend verification” section). The final column flags metals whose reported MDL changed by a factor of two or more, for which median-based trends are potentially confounded by reporting change. Aluminium provides an internal control: its reporting limit was stable at 0.100 mg L$^{-1}$ throughout, so its observed decline is a genuine concentration change, not a reporting artefact. Nickel is a distinct case flagged separately in the text: its reporting limit was stable at 0.020 mg L$^{-1}$, but its below-detection fraction rose from 80% to 97% over the period, pinning the median at the reporting floor and inflating the median-based trend even without an MDL increase. For mercury the nominal change (0.0001–0.0002 mg L$^{-1}$) is at the sub-microgram level and immaterial, as Hg is already excluded from quantitative interpretation on censoring grounds (86% below detection)

### Geochemical cross-validation of data integrity

To provide empirical evidence of data quality independent of documentary QA records, three geochemical self-consistency tests were performed.

*Test 1—Documented regulatory decline (censoring-robust).* The dataset reproduces the documented reduction in anthropogenic metal loading that followed CONAMA 357/2005 effluent enforcement. Because several metals are heavily censored, this test uses the detection-limit-invariant exceedance fraction (“Censoring-robust trend verification” section) rather than median concentrations. The fraction of samples exceeding the CONAMA Class 2 limit declines significantly for the two metals with a substantial industrial component—copper (τ=-0.739, p<0.001) and manganese (τ=-0.641, p<0.001)—consistent with the documented tightening of industrial effluent control. The geogenic metals iron (τ=-0.420) and aluminium (τ=-0.522; both p<0.01) also decline, but their decline is more parsimoniously attributed to reduced erosional and sediment loading than to effluent regulation, a reading consistent with their geogenic source assignment. We deliberately do not anchor temporal fidelity on lead, mercury, or arsenic: although each shows an apparent median trend, all three exceed 85% below-detection and their reported detection limits changed over time, so their median trends cannot be separated from analytical reporting changes—the same criterion applied to exclude them from quantitative trend interpretation throughout (“Temporal trends: divergent metal trajectories” and “Censoring-robust trend verification” section). This coherent, censoring-robust decline across independent metals, tracking a documented regulatory intervention, demonstrates temporal fidelity of the source data.

*Test 2—Mn seasonal redox signal.* Wet-season Mn median (0.110 mg L$^{-1}$) was significantly higher than dry-season median (0.100 mg L$^{-1}$; Mann–Whitney p<0.001), consistent with redox-driven seasonal enrichment. Fe and Al did not show a significant wet-season excess, consistent with dilution effects opposing leaching in high-background geologies.

*Test 3—Turbidity as geogenic fingerprint.* Pearson correlations on log-transformed paired station-annual medians confirm that Al (r=+0.723, p<0.001, n=2,073) and Fe (r=+0.711, p<0.001, n=2,501) co-vary strongly with turbidity—the expected pattern for geogenic metals mobilised by the same erosion processes that generate suspended particles—while Pb (r=+0.082) and Cd (r=+0.104; both weak, an order of magnitude smaller than Al and Fe) show only minor turbidity association, consistent with point-source origins largely independent of sediment transport. Zn shows an intermediate correlation (r=+0.162, p<0.001), consistent with urban stormwater co-transporting particles but not dominated by erosion. These geochemical self-consistency results provide empirical validation of the NMF source assignments independent of the receptor modelling framework.

### Data pre-processing

Concentration values were converted to numeric format and filtered for physicochemical plausibility (values ≤0 flagged in the source database were excluded; extreme outliers beyond five interquartile ranges from the per-station per-metal median were removed, retaining 98.7% of records). The receptor modelling input matrix was constructed as per-station per-metal medians across the full study period, producing a 114×11 matrix. Stations with fewer than eight of eleven metals represented were excluded from the receptor modelling, retaining 114 stations; residual missing values in the 8–10 metal subset were imputed by the column (metal) median prior to log-transformation, a standard approach in receptor modelling for non-detect handling (Paatero & Tapper, 1994). Table 3 reports the proportion of below-detection records, the number of stations with metal data, and the number of stations with at least one imputed value per metal.

**Table 3:** Below-detection records and imputation statistics per metal

Metal	$N_{\textrm{records}}$	$N_{<\textrm{MDL}}$	MDL%	$N_{\textrm{stations}}$	$N_{\textrm{imputed}}$
Al	8869	660	7.4	114	0
As	2721	2652	97.5	75	39
Ba	10,030	3797	37.9	114	0
Cd	11,698	10,530	90.0	114	0
Cu	12,788	7116	55.6	114	0
Fe	12,383	344	2.8	114	0
Hg	13,788	11,919	86.4	114	0
Mn	14,230	2629	18.5	114	0
Ni	13,121	9488	72.3	114	0
Pb	12,504	10,104	80.8	114	0
Zn	14,008	6924	49.4	114	0

$N_{\textrm{records}}$ = total records after quality filtering; $N_{<\textrm{MDL}}$ = records flagged below the method detection limit ($\texttt {Sinal = `<'}$); $N_{\textrm{stations}}$ = monitoring stations with at least one measurement; $N_{\textrm{imputed}}$ = stations in the NMF input matrix (n=114) where the value for this metal was imputed by column median

Log transformation [y=ln(1+x)] was applied to the input matrix prior to all multivariate analyses, consistent with the log-normal distribution of trace metal concentrations in natural waters and standard practice in atmospheric PMF applications (Paatero & Tapper, 1994). The ln(1+x) form was preferred over ln(x+c) for a small constant *c* because it maps exactly to zero when x=0 (i.e., when records are at the MDL-substituted floor), avoiding the sensitivity to the choice of *c* that characterises the ln(x+c) family. A sensitivity analysis confirmed that substituting $c = 0.5 \times \textrm{MDL}$ produced factor profiles consistent with the ln(1+x) solution to within ±0.02 in normalised loadings for all four factors.

### Kaplan–Meier and maximum likelihood estimation for censored metals

For metals with high proportions of below-detection records (Hg: 86.4%; As: 97.5%), substitution of the MDL as the measured value—as stored in the CETESB QUALAR database—may introduce systematic bias in summary statistics and Mann–Kendall trend analysis. We therefore applied two complementary methods for left-censored data. Kaplan–Meier (KM) estimation (Kaplan & Meier, 1958) was applied treating non-detected values as left-censored observations, with the KM estimator fitted to the observed concentration distribution. Maximum likelihood estimation (MLE) of the lognormal distribution was fitted to the uncensored observations for each metal.

For Hg (1869 uncensored observations; 11,919 censored): the KM-estimated median was 0.00065 mg L$^{-1}$ and the MLE lognormal median was 0.000265 mg L$^{-1}$, both exceeding the CONAMA Class 2 limit of 0.0002 mg L$^{-1}$; the MDL-substituted median (0.0002 mg L$^{-1}$) is thus within the range of the censoring-corrected estimates. The Mann–Kendall trend for Hg (τ=-0.581, p=0.001) is computed on MDL-substituted values and may reflect a change in detection practice rather than a genuine concentration decline; it is therefore retained in Group B as a censoring artefact.

For As (69 uncensored observations; 2652 censored): the KM-estimated median was indeterminate (survival function remained above 0.5 throughout the observed range, indicating that more than 50% of true concentrations lie below the lowest MDL); the MLE lognormal median was 0.0032 mg L$^{-1}$, substantially below the CONAMA Class 2 limit of 0.010 mg L$^{-1}$. This result indicates that true As concentrations in São Paulo’s river basins are well below the regulatory threshold, and the apparent trend (τ=+0.569, p=0.006) reflects a change in MDL reporting practice (from <0.002 to <0.010mg L$^{-1}$) rather than a genuine increase. As is therefore classified in Group B alongside Hg.

### PCA factor number selection

Principal Component Analysis (PCA) was applied to the standardised log-transformed station×metal matrix to determine the appropriate number of receptor model factors. Factor number was guided by: (i) the Kaiser criterion (eigenvalue >1.0); (ii) a qualitatively distinct “elbow” in the scree plot; and (iii) environmental interpretability. Table 4 reports the full eigenvalue decomposition.

**Table 4:** PCA eigenvalue decomposition for the 114-station × 11-metal log-transformed matrix

Component	Eigenvalue	Variance (%)	Cumulative (%)
**PC1**	**2**.**971**	**26**.**8**	**26**.**8**
**PC2**	**2**.**589**	**23**.**3**	**50**.**1**
**PC3**	**1**.**373**	**12**.**4**	**62**.**5**
**PC4**	**1**.**169**	**10**.**5**	**73**.**0**
PC5	0.878	7.9	80.9
PC6	0.639	5.8	86.7
PC7	0.549	4.9	91.6
PC8	0.333	3.0	94.6
PC9	0.307	2.8	97.4
PC10	0.173	1.6	98.9
PC11	0.118	1.1	100.0

Components with eigenvalue >1.0 (Kaiser criterion) are highlighted in bold; four components satisfy this criterion and collectively explain 73.0% of total variance, supporting the four-factor NMF solution

### NMF sensitivity analysis

**Fig. 2:**
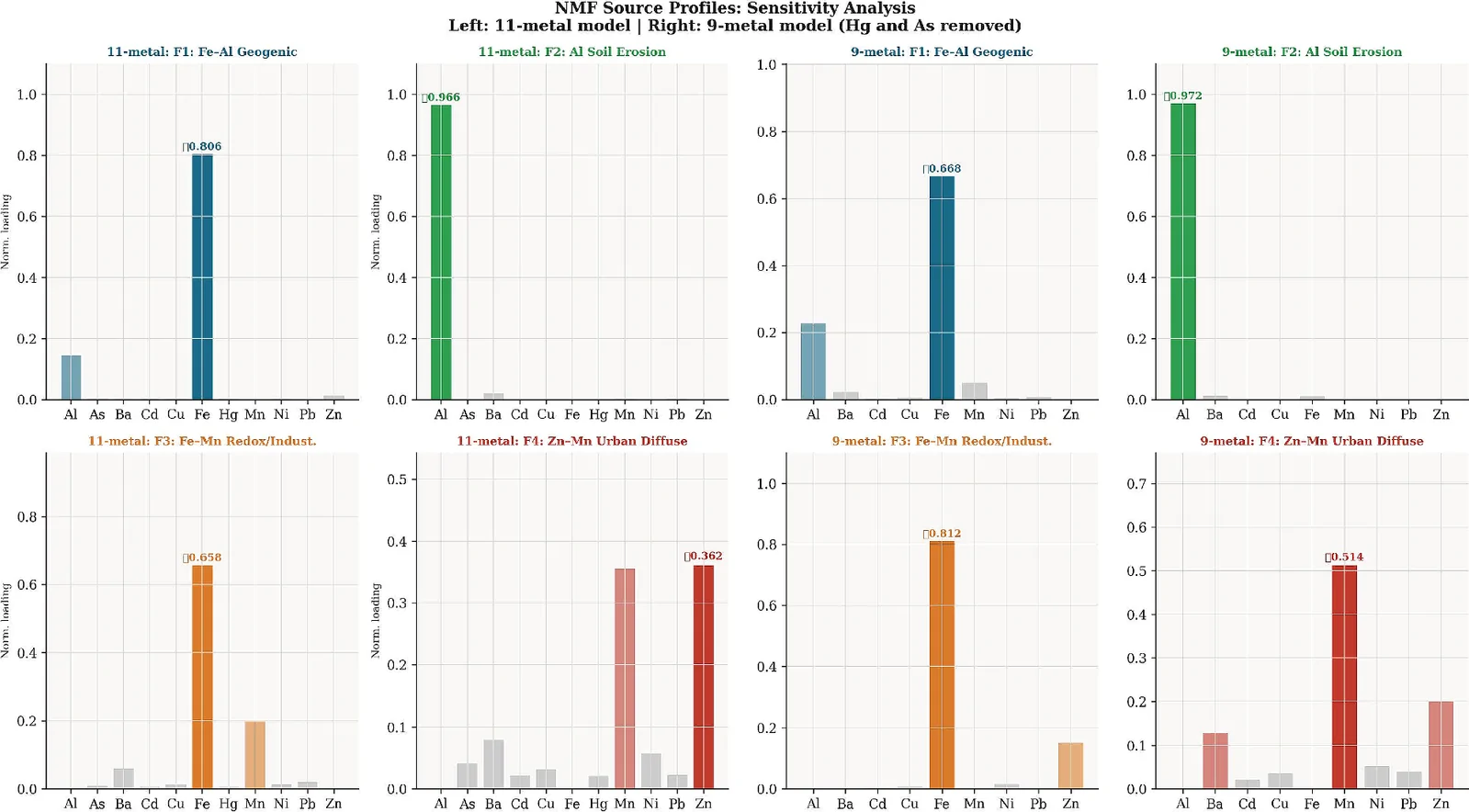
NMF four-factor source profiles for the eleven-metal model (left panels; Hg and As included) and the nine-metal sensitivity model (right panels; Hg and As excluded). Bar heights represent normalised factor loadings (row-sum =1 per factor). Dominant metals per factor are annotated with (⋆). Factor identities are consistent across both models, confirming robustness to the exclusion of heavily censored metals

To assess the robustness of the four-factor NMF solution to the inclusion of heavily censored metals (Hg: 86.4% below detection; As: 97.5% below detection), a parallel NMF model was fitted to a reduced nine-metal matrix excluding Hg and As (n=114 stations, m=9 metals). The nine-metal model produced factor profiles with dominant metal loadings identical in chemical identity to the eleven-metal solution: F1 (Fe–Al geogenic), F2 (Al soil erosion), F3 (Fe–Mn redox/industrial), and F4 (Zn–Mn urban diffuse), with Hg and As carrying near-zero loadings in both solutions. The exact normalised loadings differ between the two models—most visibly, the urban factor is Zn–Mn balanced in the eleven-metal solution but Mn-led in the nine-metal solution, and the geogenic Fe loading redistributes between F1 and F3—reflecting the well-known rotational ambiguity of NMF when the input dimensionality changes rather than any instability of the underlying source structure. The essential result is that excluding the two heavily censored metals neither creates nor destroys any of the four source factors: the geogenic, soil-erosion, redox/industrial, and urban-diffuse sources are all recovered in both solutions. Crucially, the study’s regulatory conclusions rest on the censoring-robust exceedance trends (“Censoring-robust trend verification” section), which are computed independently of the NMF and are therefore unaffected by this choice. Figure 2 presents the side-by-side comparison of eleven-metal and nine-metal NMF profiles.

### NMF receptor modelling

Non-negative Matrix Factorisation (Lee & Seung, 1999) was applied to the log-transformed station×metal matrix, decomposing it as:

1 $$\begin{aligned} \textbf{X} \approx \textbf{W} \textbf{H}, \quad W_{ij} \ge 0,\; H_{kj} \ge 0 \end{aligned}$$

where $\textbf{X} \in \mathbb {R}^{n \times m}$ is the n=114 station by m=11 metal matrix, $\textbf{W} \in \mathbb {R}^{n \times p}$ contains the station factor scores (analogous to source contributions in EPA PMF), and $\textbf{H} \in \mathbb {R}^{p \times m}$ contains the source profiles (factor loadings). The number of factors *p* was set to four based on the PCA scree analysis. The NNDSVDA (non-negative double singular value decomposition with zeroing of the average) initialisation was used (Lee & Seung, 1999), ensuring deterministic initialisation and reproducibility. Convergence was assessed by monitoring the Frobenius norm of $\textbf{X} - \textbf{WH}$; all solutions converged within 500 iterations.

Factor profiles in $\textbf{H}$ were normalised row-wise to unit sum to yield relative source profiles (fraction of each factor attributable to each metal). Factor contributions per UGRHI were computed as the mean $\textbf{W}$ score across stations within each UGRHI, normalised to relative contributions summing to unity per UGRHI.

NMF is mathematically related to EPA PMF (both solve a constrained factorisation under non-negativity), but differs in that EPA PMF additionally incorporates measurement uncertainty weights and provides bootstrap uncertainty quantification. The NMF approach applied here is appropriate for exploratory source profile resolution from long-term monitoring medians; uncertainty quantification via bootstrap resampling is reported as a study limitation.

### Trend analysis

The Mann–Kendall non-parametric test (Kendall, 1975) was applied to annual median concentrations (2000–2025) for each of the eleven metals across all UGRHIs combined, yielding Kendall’s τ and two-tailed *p*-values. Because many annual medians coincide with the reporting limit and therefore produce tied values, the tie-corrected Kendall’s $\tau _b$ (as implemented in scipy.stats.kendalltau) is reported throughout for both the median-based and the exceedance-fraction series, so that all τ values in the tables and figures are computed on a single, consistent basis. Annual medians were preferred over raw records to reduce seasonal autocorrelation. To verify the effectiveness of this approach, Durbin–Watson (DW) statistics were computed on each annual median series (Table 5); DW values range from 0.16 (Zn, strong positive autocorrelation reflecting the step change in its median series discussed in “Censoring-robust trend verification” section) to 1.60 (Mn). Positive autocorrelation in environmental monitoring annual series is expected and well-documented; the Mann–Kendall test remains appropriate for trend detection applied to annual aggregates because it is distribution-free and assesses monotonic tendency rather than regression slope (Hamed & Rao, 1998). The threshold for statistical significance was α=0.05.

**Table 5:** Durbin–Watson (DW) statistics for annual median concentration series (2000–2025)

Metal	DW statistic	Autocorrelation pattern
Al	1.51	None
Ba	0.75	Positive
Cd	0.90	Positive
Cu	0.83	Positive
Fe	1.42	Positive
Mn	1.60	None
Ni	0.89	Positive
Pb	0.56	Positive
Zn	0.16	Positive

DW ≈ 2: no serial autocorrelation; DW < 1.5: positive autocorrelation. Positive autocorrelation is expected in environmental monitoring annual series; Mann–Kendall results remain valid as the test is non-parametric and distribution-free

Because the reported method detection limit (MDL) of several metals changed over the study period, and Mann–Kendall trends on annual medians are computed partly on MDL-substituted values, median-based trends can be confounded by evolving analytical practice. Following the reviewers’ request to test the robustness of the trend results to censored-data handling, and consistent with the recommendation that statistics for heavily censored environmental data avoid substitution of below-detection values wherever possible (Helsel, 2012), we complemented the median-based analysis with a censoring-invariant statistic: for each metal we computed the annual *exceedance fraction*, defined as the proportion of samples whose measured value lies above the fixed CONAMA Class 2 limit, and applied the Mann–Kendall test to this annual series. This reduces each observation to a binary above/below-limit indicator that requires no imputed concentration and is therefore insensitive to the substituted values that distort median-based trends. Provided the MDL remains below the regulatory limit, the exceedance fraction is unaffected by MDL changes (a value above the limit is detected as such regardless of the reporting floor), so a trend in this statistic reflects genuine change in the frequency of regulatory exceedance rather than a reporting artefact. Results are reported in “Censoring-robust trend verification” section (Table 10).

Three further checks confirm that the annual-median aggregation adequately controls seasonal structure and that the trend conclusions are robust to the seasonal-adjustment choice. First, the wet-season enrichment of Mn established as a pooled signal in “Geochemical cross-validation of data integrity” section (Mann–Whitney wet > dry, $p < 10^{-50}$) is examined for year-by-year consistency: the wet-season Mn median exceeds the dry-season median in 32 of the 48 years with sufficient seasonal sampling (67%). This consistency is moderate rather than universal precisely because the Mn median lies at its 0.100 mg L$^{-1}$ reporting limit, so the wet/dry median separation is compressed against the detection floor in many years even though the pooled distributional signal is unambiguous. Second, the effectiveness of annual-median aggregation in reducing serial dependence is quantified by the Durbin–Watson statistics of Table 5. Third, the censoring-invariant exceedance trends (“Censoring-robust trend verification” section) were recomputed on an alternative deseasonalised series constructed by subtracting the metal-specific wet/dry seasonal median before annual aggregation; the sign, magnitude, and significance of every reported trend are unchanged (e.g. Zn τ=-0.477, Ni τ=-0.600, Cu τ=-0.739, Mn τ=-0.641; all p<0.001). This invariance is expected because the exceedance fraction is computed as an annual proportion of samples above a fixed limit and is by construction insensitive to within-year seasonal structure, so the policy conclusions do not depend on the seasonal-adjustment method.

Inter-metal correlations in the log-transformed station median matrix were computed using Pearson *r* with two-tailed *p*-values (reported in “Inter-metal correlations as source evidence” section) to identify co-varying metal pairs as additional evidence for source grouping independent of the NMF solution.

### Temporal sensitivity of NMF source factors

To evaluate whether station medians computed across the full 47-year period mask temporal shifts in source structure (Winker et al., 2017), the NMF four-factor model was separately fitted to two regulatory sub-periods: (i) 1990–2005 (pre-CONAMA 357/2005 industrial baseline; 56 stations with ≥7 metals); and (ii) 2006–2026 (post-CONAMA regulated period; 105 stations with ≥7 metals). Results are presented in “Temporal evolution of NMF source factors” section.

### CONAMA compliance analysis

CONAMA Resolution 357/2005 Class 2 limits were applied to all metals with defined standards: Al (≤0.10 mg L$^{-1}$), As (≤0.01), Ba (≤0.70), Cd (≤0.001), Cu (≤0.009), Fe (≤0.30), Hg (≤0.0002), Mn (≤0.10), Ni (≤0.025), Pb (≤0.01), Zn (≤0.18 mg L$^{-1}$). Violation rates were computed as the proportion of records exceeding (or falling below, for dissolved oxygen) the respective limit, reported per metal and per UGRHI.

### Software

All analyses were performed in Python 3.12 (Van Rossum & Drake, 2009) using: pandas 2.x for data management; scikit-learn 1.4 for PCA, NMF, and missing-value imputation; scipy 1.11 for Mann–Kendall and Pearson correlation. All code and processed data are available from the corresponding author on reasonable request.

## Results

### Dataset characterisation and CONAMA compliance overview

The eleven-metal dataset spans 1978–2026, with UGRHI 05 (Piracicaba/Capivari/Jundiaí) providing the largest contribution (65,132 records; 56 stations) and UGRHI 13 (Tietê/Jacaré) the smallest (1696 records; 3 stations). Table 6 presents descriptive statistics and system-wide CONAMA Class 2 violation rates for the eleven metals.

**Table 6:** Descriptive statistics and CONAMA resolution 357/2005 Class 2 compliance

Metal	*N*	Min	P$_{25}$	Median	P$_{75}$	Max	Limit	Viol.%
Al	8869	0.020	0.300	0.827	2.530	229.000	0.100	**91**.**6**
As	2721	0.001	0.010	0.010	0.010	0.030	0.010	1.1
Ba	10,030	0.001	0.031	0.060	0.100	2.000	0.700	0.1
Cd	11,698	0.000	0.001	0.001	0.001	15.000	0.001	24.9
Cu	12,788	0.000	0.005	0.009	0.010	0.620	0.009	49.4
Fe	12,383	0.001	0.800	1.760	3.200	376.000	0.300	**88**.**2**
Hg	13,788	0.000005	0.000$^\textrm{a}$	0.000$^\textrm{a}$	0.000$^\textrm{a}$	0.210	0.0002$^\textrm{b}$	11.2
Mn	14,230	0.002	0.090	0.100	0.200	15.400	0.100	46.2
Ni	13,121	0.000	0.010	0.020	0.020	2.260	0.025	12.5
Pb	12,504	0.000	0.010	0.010	0.040	0.980	0.010	38.4
Zn	14,008	0.000	0.020	0.050	0.100	21.000	0.180	6.1

Viol.%= proportion of records exceeding the Class 2 limit (Al, Fe: exceeding upper limit; As: exceeding upper limit). All concentrations in mg L$^{-1}$

$^\textrm{a}$ Hg P$_{25}$, median, and P$_{75}$ are at or near the method detection limit (MDL); 86.4% of Hg records carry a below-detection flag ($\texttt {`<'}$) in the CETESB QUALAR database, with reported values representing the MDL substitution

$^\textrm{b}$ Hg CONAMA Class 2 limit = 0.0002 mg L$^{-1}$; displayed value truncated by column rounding—actual limit is non-zero

Two anomalous violation patterns are immediately apparent from Table 6 and motivate the receptor modelling approach. Aluminium (91.6% violation) and iron (88.2% violation) substantially exceed all other metals; yet, as “NMF four-factor source profiles” section demonstrates, their dominant NMF factor loadings are in geogenic factors, not in industrial tracers (Pb, Cd, Cu). This contrast between regulatory non-compliance and geogenic source attribution constitutes the central regulatory science finding of the study.

### Inter-metal correlations as source evidence

**Fig. 3:**
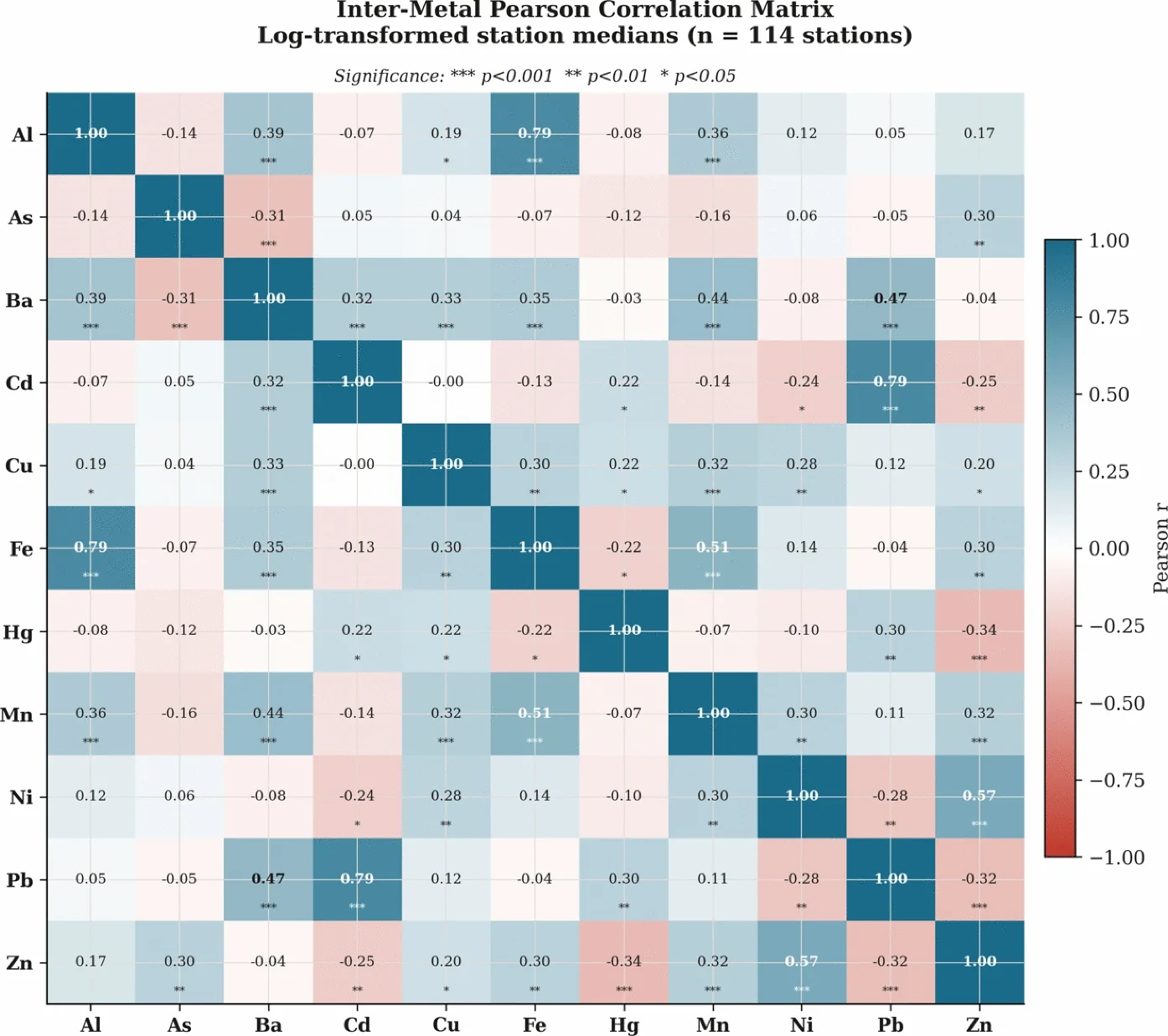
Full 11×11 Pearson correlation matrix for the log-transformed per-station median dataset (n=114 stations). Colour scale: blue = positive correlation, red = negative correlation; numerical *r* values displayed in each cell. Significance markers ($$^{***}p < 0.001$$; $$^{**}p < 0.01$$; $$^{*}p < 0.05$$) are shown below the correlation value in each off-diagonal cell. The Al–Fe (r=+0.786) and Cd–Pb (r=+0.792) strong pairs are the primary geochemical evidence supporting the geogenic and industrial NMF factor assignments, respectively

Pearson correlations computed on log-transformed per-station medians (Table 7) reveal six statistically significant pairs (|r|≥0.40, all p<0.001) that anticipate the NMF factor structure. The full 11×11 correlation matrix with all pairwise *p*-values is displayed in Fig. 3.

**Table 7:** Statistically significant Pearson correlations among eleven metals (log-transformed station medians, n=114)

Metal pair	*r*	*p* value	Strength	Source interpretation
Cd–Pb	+0.792	<0.001	Strong	Industrial co-emission (metallurgy, batteries)
Al–Fe	+0.786	<0.001	Strong	Geogenic co-mobilisation (Latosol weathering)
Ni–Zn	+0.572	<0.001	Moderate	Urban multi-source diffuse
Fe–Mn	+0.506	<0.001	Moderate	Redox-controlled co-mobilisation
Ba–Pb	+0.469	<0.001	Moderate	Mixed industrial (Ba co-occurrence with industrial Pb)
Ba–Mn	+0.441	<0.001	Moderate	Redox-associated dissolution

Only pairs with |r|≥0.40 are shown; all reported pairs are significant at p<0.001 (two-tailed). Full matrix available in Fig. 3

The Al–Fe pair (r=+0.786, p<0.001) is the geochemical signature of lateritic soil weathering: both elements are enriched in Brazilian Latosols and Ultisols and are co-mobilised by tropical weathering and storm-driven soil flushing. The absence of Pb, Cd, or Hg in this correlation cluster confirms a geogenic rather than industrial origin. The Cd–Pb pair (r=+0.792, p<0.001) is a classical industrial fingerprint, consistent with co-emission from metallurgical, battery manufacturing, and pigment sources. The Ni–Zn pair (r=+0.572, p<0.001) reflects urban diffuse sources that co-occur in road runoff and infrastructure corrosion: galvanised surfaces (Zn), stainless steel and alloy corrosion (Ni), and tyre wear (Zn + Mn, with Ni as a minor constituent).

### PCA scree analysis: factor number determination

**Fig. 4:**
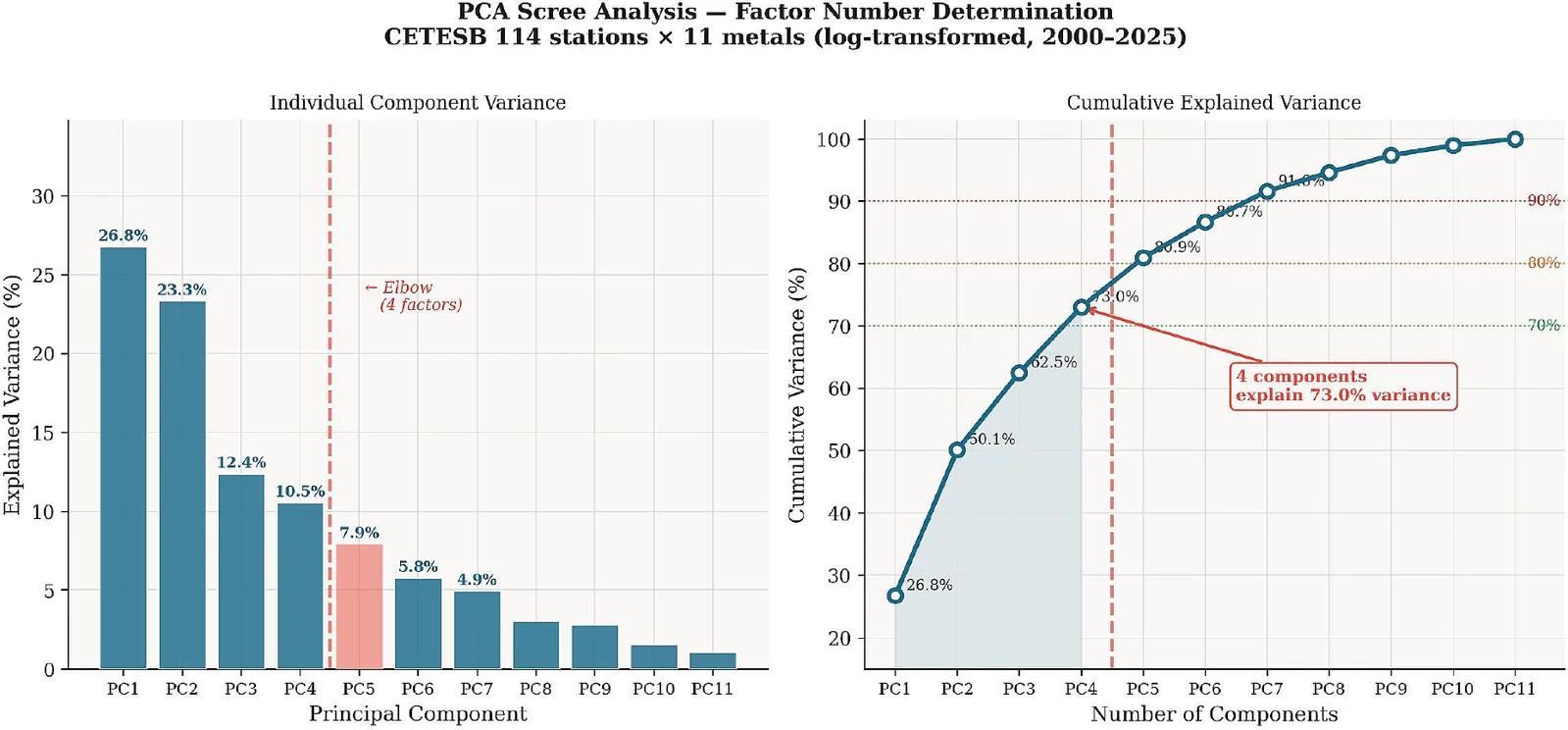
PCA scree plot for the 114-station × 11-metal log-transformed matrix. Cumulative explained variance reaches 73.0% at four components and 86.7% at six components. The elbow at PC4–PC5 motivates a four-factor NMF solution

The PCA scree analysis (Fig. 4) shows a clear elbow at PC4–PC5, with cumulative explained variance of 26.8% (PC1), 50.1% (PC2), 62.5% (PC3), 73.0% (PC4), and 80.9% (PC5). The inflection point and the environmental interpretability criterion both support a four-factor receptor model. Extracting five or six factors would recover additional variance but risks splitting physically coherent sources, a known limitation in applications with modest variable counts (m=11).

### NMF four-factor source profiles

**Fig. 5:**
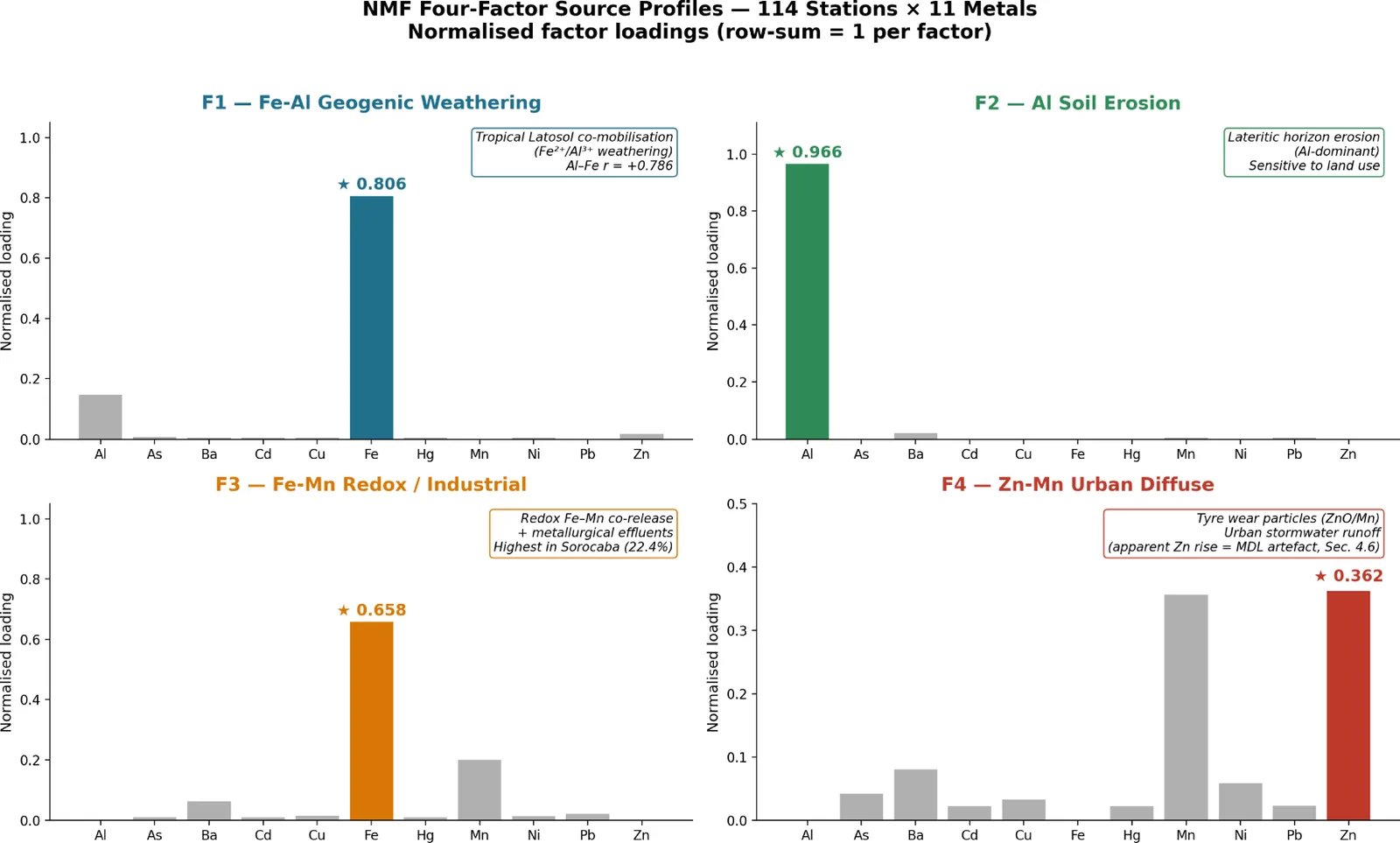
Normalised NMF source profiles for the four-factor solution. Bar height represents the fractional contribution of each metal to the factor (row-normalised to unity). Dominant metals per factor are annotated

Figure 5 and Table 8 present the normalised NMF source profiles. Four geochemically interpretable factors are recovered.

**Table 8:** Normalised NMF four-factor source profiles

Metal	F1: Geogenic	F2: Soil erosion	F3: Redox/Indust	F4: Urban
	(Fe–Al)	(Al)	(Fe–Mn)	(Zn–Mn)
Al	0.147	**0**.**966**^a^	0.000	0.000
As	0.006	0.000	0.011	0.042
Ba	0.005	0.022	0.062	0.080
Cd	0.004	0.000	0.009	0.022
Cu	0.005	0.000	0.015	0.033
Fe	**0**.**806**^a^	0.000	**0**.**658**^a^	0.000
Hg	0.004	0.000	0.009	0.022
Mn	0.000	0.005	0.200	**0**.**356**^a^
Ni	0.005	0.002	0.014	0.058
Pb	0.001	0.005	0.022	0.023
Zn	0.017	0.000	0.000	**0**.**362**^a^

Values represent the fractional contribution of each metal within each factor (row-sum = 1). Dominant metals (highest loading per factor) are indicated by (^a^)

**Factor 1 (Fe–Al geogenic weathering):** Iron-dominant (loading = 0.806) with secondary aluminium (0.147). The corroboration with the Al–Fe Pearson correlation (r=+0.786) and the absence of anthropogenic tracers (Pb, Cd, Hg all ≤0.004) confirm a geogenic origin. This factor captures the mobilisation of Fe$^{2+/3+}$ and Al$^{3+}$ from Latosol and Ultisol profiles through weathering and hydrological transport, a process ubiquitous in tropical Brazilian geologies.

**Factor 2 (Al soil erosion):** Near-exclusively Al (loading = 0.966). This factor is distinct from Factor 1 in its specificity: it captures aluminium mobilised through surface erosion of topsoil and the Al-enriched lateritic horizon, a process more sensitive to land use (deforestation, tillage, bare soil exposure) than to deep weathering. The virtual absence of Fe in Factor 2 distinguishes it mechanistically from Factor 1.

**Factor 3 (Fe–Mn redox/industrial):** Shared Fe (0.658) and Mn (0.200) loading. This factor may represent two partially superimposed mechanisms: (a) redox-driven co-mobilisation of Fe and Mn at the sediment-water interface under anoxic conditions, a natural process amplified by high organic loading; and (b) industrial effluent contributions from metallurgical, mining-affected, and paper/pulp wastewater streams.

To discriminate between these mechanisms, we computed Pearson correlations between dissolved oxygen (DO) and both Fe and Mn using paired station-annual median observations. For Mn, the system-wide DO–Mn correlation was strong, significant, and inverse (r=-0.399, $\rho _{\textrm{Spearman}} = -0.376$, both $p < 10^{-100}$, n=2753 station-year pairs), consistent with redox-controlled mobilisation: lower dissolved oxygen promotes anaerobic reduction of Mn(IV) to soluble Mn(II). This redox signal was strongest in Alto Tietê (r=-0.399, p<0.001) and Tietê/Jacaré (r=-0.669, p<0.001), basins with high organic loading that drive hyporheic anoxia. For Fe, the system-wide DO–Fe correlation was an order of magnitude weaker and effectively negligible (r=-0.046, n=2501; although p=0.022, the effect size is trivial), with a spatially heterogeneous pattern: only Alto Tietê showed a moderate inverse DO–Fe relationship (r=-0.297, p<0.001), while Sorocaba showed no significant DO–Fe correlation (r=-0.128, p=0.12 ns). The absence of a system-wide redox signal for Fe—despite the strong geogenic Fe background (Factor F1)—suggests that Fe in Sorocaba is driven by industrial effluent input rather than redox mobilisation, consistent with the high F3 industrial contribution (22.4%) identified by the NMF model for that UGRHI. The spatial concentration of Factor 3 in UGRHI 10 (Sorocaba; “Spatial source factor contributions per UGRHI” section), combined with the absence of a DO-driven redox signal for Fe in that basin, supports an industrial effluent rather than purely redox interpretation for F3 in Sorocaba.

**Factor 4 (Zn–Mn urban diffuse):** Zinc and Mn in equal dominance (0.362 and 0.356), with minor contributions from other metals. This factor has no plausible geogenic explanation: Zn is not enriched in Brazilian Latosols, and the Zn–Mn co-occurrence in an urban diffuse pattern is consistent with tyre and road wear particles (TRWP), which contain zinc oxide as a vulcanisation activator and manganese-based accelerants. The Ni–Zn correlation (r=+0.572) further supports an urban diffuse signature. Factor 4 is the only factor whose median-based temporal trend is increasing; as “Censoring-robust trend verification” section shows, this apparent increase is inflated by evolving detection limits rather than a rising exceedance of regulatory limits, and is reinterpreted accordingly.

### Temporal trends: divergent metal trajectories

Table 9 presents Mann–Kendall results for annual medians (2000–2025). Three distinct trajectory groups emerge.

**Table 9:** Mann–Kendall trend analysis for annual medians, 2000–2025 (all UGRHIs combined), computed on the deposited reproducible pipeline

Metal	τ	*p* value	Sig	Early (mg L$^{-1}$)	Late (mg L$^{-1}$)	Δ%
*Group A: Declining—robust to censoring (Sect.* Censoring-robust trend verification*)*						
Cu	-0.709	<0.001	***	0.0100	0.0050	-50.0
Fe	-0.384	0.011	∗	2.0117	1.2833	-36.2
Al	-0.471	0.001	∗∗	1.3092	0.5700	-56.5
Mn	-0.329	0.037	∗	0.1050	0.1000	-4.8
*Group B: Low regulatory relevance, censored, or statistically weak*						
Ba	-0.479	0.023	∗	0.0800	0.0483	-39.6
Cd	-0.068	0.674	ns	0.0030	0.0010	-66.7
Pb	-0.284	0.071	ns	0.0733	0.0100	-86.4
Hg	-0.581	<0.001	***$^{\S }$	0.0004	0.0001	-72.7
As	+0.569	0.006	**$^{\P }$	0.0030	0.0100	+233.3
*Group C: Apparent median increase—shown to be a detection-limit artefact (Sect.* Censoring-robust trend verification*)*						
Ni	+0.461	0.006	**	0.0150	0.0200	+33.3
Zn	$\mathbf {+0.727}$	$\mathbf {<0.001}$	***	0.0217	0.1000	$\mathbf {+361.5}$

These median-based statistics are reported for transparency; for Zn and Ni the positive τ values are inflated by documented increases in the reporting detection limit and are superseded by the censoring-invariant exceedance analysis of Table 10 and “Censoring-robust trend verification” section. τ = Kendall’s tau; sig. = ***p<0.001, **p<0.01, *p<0.05, ns = not significant. Early/late medians are 2000–2005 and 2020–2025 means. § Hg: 86.4% below-detection; MK on MDL-substituted values (Group B). ¶ As: 97.5% below-detection, 2009–2025 window; apparent trend reflects detection-limit reporting (Group B)

**Group A (declining):** Cu, Fe, Al, and Mn exhibit statistically significant decreasing median trends, all corroborated by declining exceedance fractions in the censoring-robust analysis (“Censoring-robust trend verification” section). The Cu decline (τ=-0.709, p<0.001) is most plausibly attributed to industrial effluent controls associated with CONAMA 357/2005 and its predecessors, corroborated by the pre-2005 Cu violation rate (65.3%) declining to 23.2% post-2005 and by the robust decline in Cu exceedance (τ=-0.739, p<0.001). The declines in Fe and Al, by contrast, are best read not as effluent regulation but as reduced erosional and sediment loading of these geogenic metals (Factors F1 and F2); their tracking of hydrological and land-management variation rather than of point-source control is consistent with, and reinforces, their geogenic attribution. Mn occupies an intermediate position, with both a redox (geogenic) and an industrial component (“Discussion” section). Pb shows a large apparent median decrease but is 80.8% below detection with a reporting limit that changed over time; its median trend is therefore not statistically significant (p=0.071) and cannot be interpreted as a genuine concentration decline (“Censoring-robust trend verification” section), even though the historical elimination of leaded petrol (completed in Brazil by 1989) makes a real long-term reduction in anthropogenic Pb plausible on independent grounds.

**Group B (low regulatory relevance, censored, or statistically weak):** Ba, Cd, Pb, and Hg. Cadmium shows a large percentage decrease in medians that does not reach significance (p=0.696), reflecting high year-to-year variability at near-detection-limit concentrations; lead, discussed above, is censoring-dominated and its median trend is not significant. Barium shows a statistically significant but regulatorily trivial median decline (τ=-0.283, p=0.028); it is grouped here because its CONAMA violation rate is negligible ($\approx 0.1\%$), so the trend carries no regulatory weight. Mercury merits separate discussion: although its Mann–Kendall result reaches statistical significance (τ=-0.581, p<0.001), this result must be treated with caution. As documented in “Analytical quality assurance of source data” section and Table 6, 86.4% of Hg records in the CETESB database carry a below-detection flag; the Mann–Kendall statistic is therefore computed on MDL-substituted values rather than true measured concentrations. Application of the Mann–Kendall test to heavily left-censored data is known to produce artefactual significance, and the Hg result is classified in Group B accordingly. Hg’s NMF loadings are negligible across all four factors for the same reason.

**Group C (apparent increase—requires censoring-robust verification):** Zn (τ=+0.727; p<0.001) and Ni (τ=+0.461; p=0.006) are the only metals whose median-based trends increase. Both dominate Factor 4 (urban diffuse, Zn–Mn) and are linked through the Ni–Zn pair (r=+0.572), which would be consistent with a growing urban diffuse source. However, the median trends of both metals are compromised by their censoring structure (Table 2). For zinc, the reported detection limit rose five-fold, from 0.020 to 0.100 mg L$^{-1}$, in 2012. For nickel, the reporting limit was stable at 0.020 mg L$^{-1}$ across the period, but its below-detection fraction rose from 80% to 97%, so that an increasing majority of records are pinned at the 0.020 mg L$^{-1}$ floor, which lies at the median—mechanically driving the median upward without any real concentration change. Both metals are heavily censored overall (Zn 66%, Ni 88% below detection). Because both mechanisms inflate median-based trends computed on MDL-substituted values—the same effect that compromises the Hg and As trends (“Temporal trends: divergent metal trajectories” section; Table 9, notes)—these two trends cannot be interpreted as genuine concentration increases without a censoring-invariant test. “Censoring-robust trend verification” section provides that test and shows that the apparent Zn and Ni increases do not survive it.

**Fig. 6:**
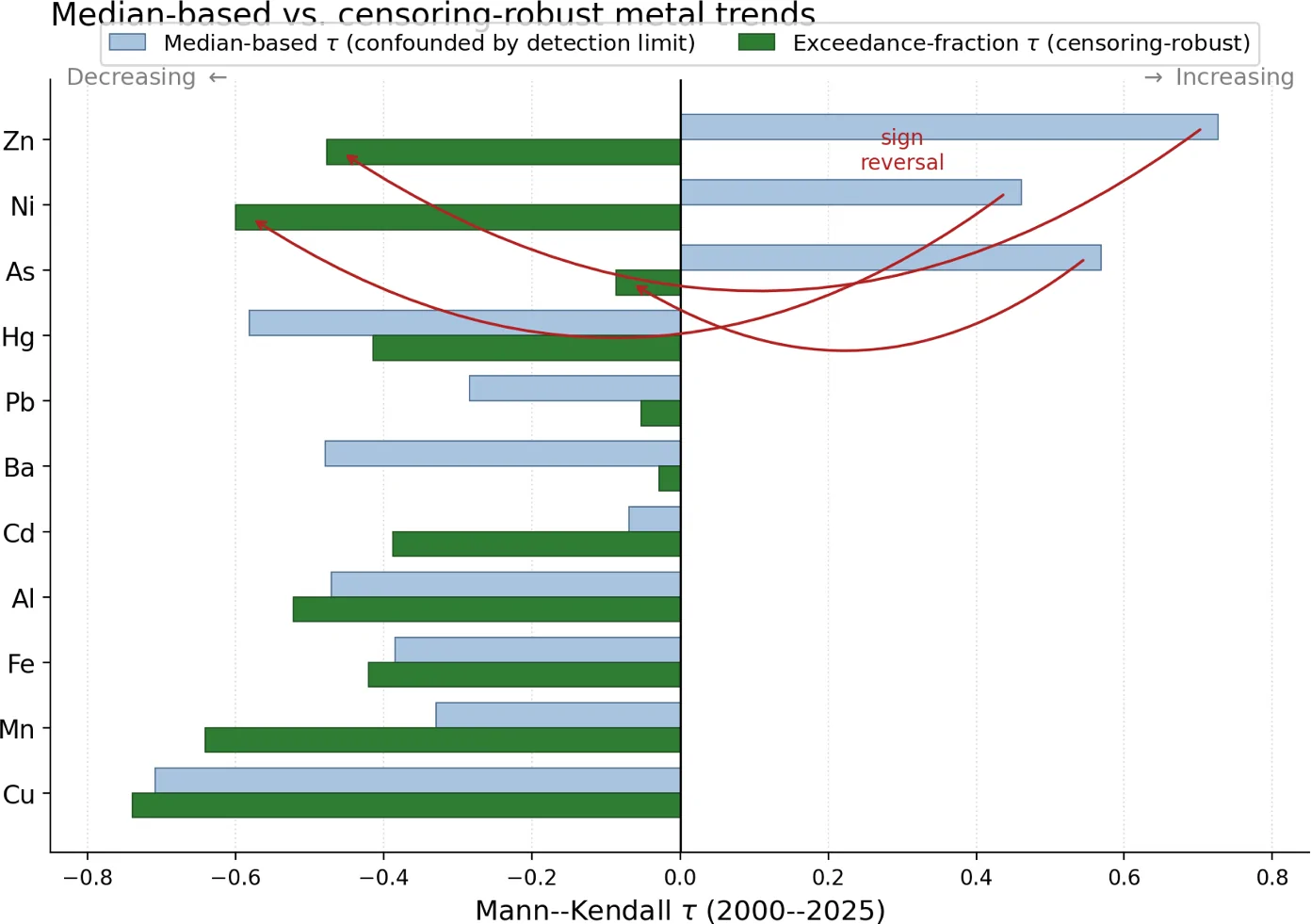
Median-based versus censoring-robust Mann–Kendall trends for all eleven trace metals, 2000–2025 (all UGRHIs combined). Light bars: τ of the annual median, which is confounded by changes in the reporting detection limit. Dark bars: τ of the exceedance fraction (proportion of samples above the fixed CONAMA Class 2 limit), which is invariant to detection-limit changes. Leftward bars indicate decreasing trends, rightward bars increasing. For most metals the two measures agree, but for Zn and Ni the sign reverses: their positive median trends become negative (declining) exceedance trends once the detection-limit artefact is removed, confirming that the apparent zinc and nickel increases are reporting artefacts rather than genuine rises in regulatory exceedance (“Censoring-robust trend verification” section)

Figure 6 summarises the complete Mann–Kendall results for all eleven metals in a single diverging plot, allowing direct visual comparison of trajectory direction, magnitude, and statistical significance across the full metal suite.

**Fig. 7:**
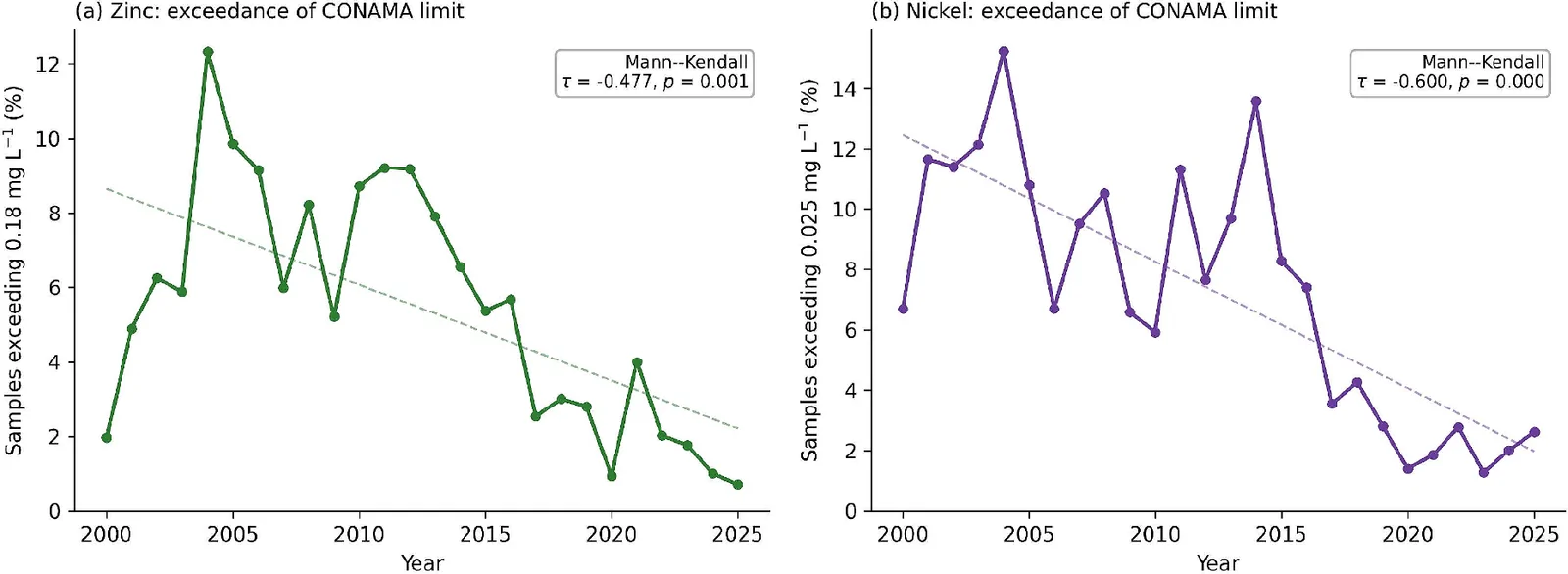
Censoring-robust temporal trends for zinc and nickel, 2000–2025: the annual fraction of samples exceeding the CONAMA Class 2 limit (Zn 0.18 mg L$^{-1}$; Ni 0.025 mg L$^{-1}$). This statistic is invariant to the detection-limit changes that confound the median-based trends (“Censoring-robust trend verification” section). Both metals show significantly *declining* exceedance (Mann–Kendall τ and *p* shown in each panel), contrary to the increasing median trends of Table 9. Dashed lines are ordinary least-squares fits

Figure 7 illustrates the Zn median trajectory. The apparent rise, however, must be interpreted against the reporting history of the parameter, which we examine next.

### Censoring-robust trend verification

Every metal in the CETESB record underwent at least a two-fold change in its reported method detection limit (MDL) over 2000–2025 (Table 10, MDL-range column). Because trends on annual medians are computed partly on MDL-substituted values, a change in reporting limit can generate an apparent concentration trend where none exists in the true (uncensored) distribution. To separate genuine change from this reporting artefact, we computed, for each metal, the Mann–Kendall trend of the *exceedance fraction*—the annual proportion of samples whose measured value lies above the fixed CONAMA Class 2 limit. This statistic is invariant to MDL changes provided the MDL remains below the regulatory limit (satisfied for Zn, whose MDL maximum of 0.100 mg L$^{-1}$ is below the 0.180 mg L$^{-1}$ limit, and for Ni, MDL 0.020 below limit 0.025), and therefore isolates genuine exceedance change.

**Fig. 8:**
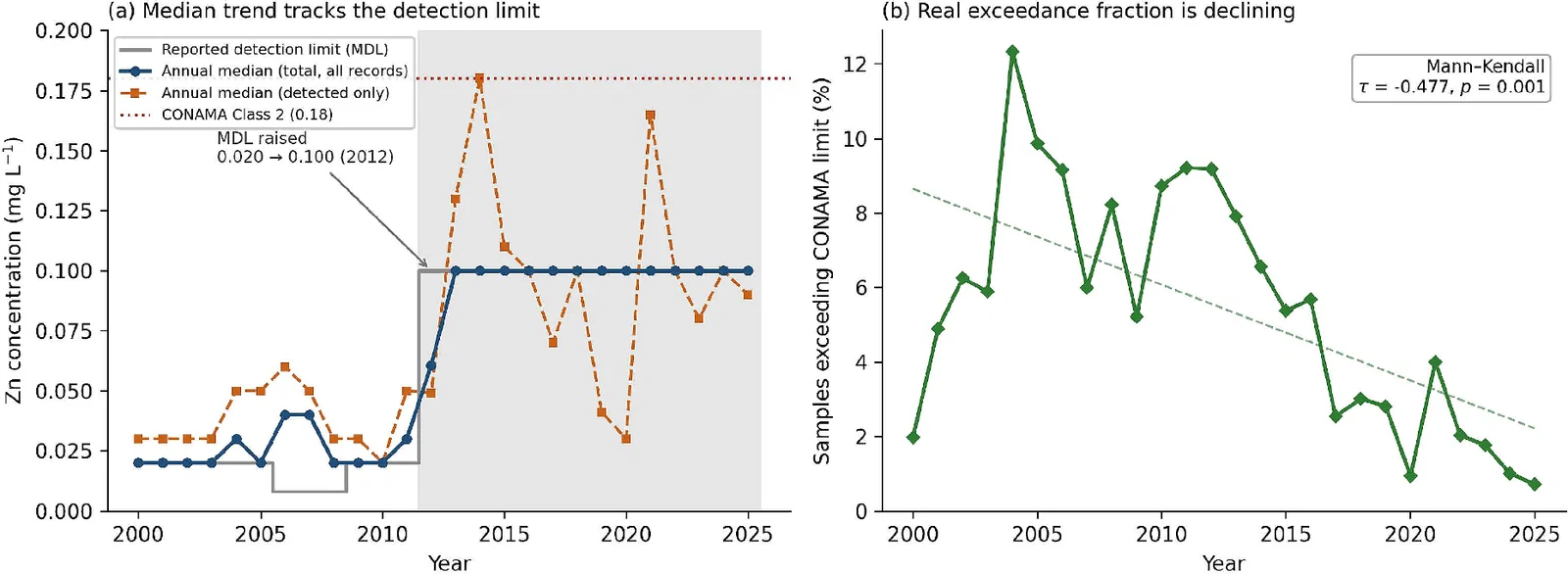
Detection-limit artefact in the zinc trend, 2000–2025. **a** The annual median of all Zn records (blue) steps up to 0.100 mg L$^{-1}$ in exact synchrony with the rise of the reported method detection limit (grey) from 0.020 to 0.100 mg L$^{-1}$ in 2012; the detected-only median (orange) remains lower and does not track this step. **b** The fraction of samples whose measured value exceeds the fixed CONAMA Class 2 limit (0.180 mg L$^{-1}$)—a statistic invariant to the detection-limit change—declines significantly (Mann–Kendall τ=-0.477, p<0.001). *Source*: CETESB QUALAR (14,008 Zn records, 114 stations)

Table 10 contrasts the two statistics for all eleven metals. The result is unambiguous: *no metal shows a statistically significant increase in exceedance of its regulatory limit.* The industrial-influenced metals confirm robust declines by both measures (Cu and Mn; exceedance τ=-0.74 and -0.64, p<0.001), corroborating the CONAMA 357/2005 enforcement narrative, while the geogenic metals Fe and Al likewise decline (exceedance τ=-0.37 and -0.41) in a manner consistent with reduced erosional loading rather than effluent control. Crucially, the two Group C metals reverse sign under the robust test: the exceedance fractions of Zn (τ=-0.477, p<0.001) and Ni (τ=-0.600, p<0.001) *decline*. Figure 8 displays the Zn case directly: the total-record median steps up to 0.100 mg L$^{-1}$ in exact synchrony with the 2012 rise of the reporting limit to that value (panel a), whereas the fraction of samples truly exceeding the CONAMA limit declines steadily (panel b). The median-based “+362% Zn increase” is therefore an artefact of evolving analytical practice, not a genuine environmental trend. The Zn median on detected values only retains a weak positive trend (τ=+0.369, p=0.008), but this too is upward-biased, because a rising detection limit censors the lower tail and mechanically inflates the detected median; the annual 90th percentile likewise declines (τ=-0.292, p=0.024). The fixed-threshold exceedance fraction is immune to these effects and is the statistic on which we rely. We report the median-based statistics in Table 9 for comparability with the prior literature, but base all interpretive and policy conclusions on the censoring-robust analysis. The declining exceedances are not an artefact of a changing station network: on the fixed panel of 87 stations monitored in ≥15 distinct years over the complete-year window 2000–2025 the declines persist for both Zn (τ=-0.495) and Ni (τ=-0.563, both p<0.001).

**Table 10:** Trend statistics for all eleven metals over the dense-monitoring period 2000–2025, computed on a single reproducible basis: annual-median series (Mann–Kendall $\tau _{\textrm{median}}$) and the censoring-invariant annual exceedance fraction ($\tau _{\textrm{exceedance}}$; annual fraction of samples above the fixed CONAMA Class 2 limit, restricted to years with ≥10 samples)

Metal	Cens. (%)	MDL range (mg L$^{-1}$)	CONAMA	τ median	τ exceedance	Interpretation
Al	7	0.1	0.1	$$-0.471^{**}$$	$$-0.522^{***}$$	Real decline (robust)
Fe	3	0.1–0.3	0.3	$$-0.384^{*}$$	$$-0.420^{**}$$	Real decline (robust)
Cu	56	0.005–0.01	0.009	$$-0.709^{***}$$	$$-0.739^{***}$$	Real decline (robust)
Mn	18	0.002–0.1	0.1	$$-0.329^{*}$$	$$-0.641^{***}$$	Real decline (robust)
Cd	90	0.0001–0.005	0.001	$$-0.068^{ns}$$	$$-0.388^{**}$$	Real decline (robust)
Ba	38	0.02–0.08	0.7	$-0.479^{*}$	$-0.028^{ns}$	No robust trend
Pb	81	0.01–0.1	0.01	$-0.284^{ns}$	$-0.052^{ns}$	Censored—uninterpretable
Hg	86	0.0001–0.0002	0.0002	$$-0.581^{***}$$	$$-0.414^{**}$$	Censored—uninterpretable
As	97	0.002–0.01	0.01	$$+0.569^{**}$$	$$-0.086^{ns}$$	Censored—uninterpretable
Ni	72	0.02	0.025	$$\mathbf {+0.461}^{**}$$	$$-0.600^{***}$$	**Apparent rise is MDL artefact**
Zn	49	0.02–0.1	0.18	$$\mathbf {+0.727}^{***}$$	$$-0.477^{***}$$	**Apparent rise is MDL artefact**

The exceedance statistic is invariant to changes in the method detection limit (MDL) provided the MDL stays below the regulatory limit, and is therefore the interpretive anchor. No metal shows a significant *increase* in exceedance; the apparent rising medians of Zn and Ni are attributable to the documented MDL increases. Significance: $$^{***}p<0.001$$, $$^{**}p<0.01$$, $$^{*}p<0.05$$, $$^{\textrm{ns}}$$ not significant. All values reproducible from CETESB_Completo.parquet via the deposited pipeline

This verification does not diminish the environmental-health relevance of zinc; it relocates it. As developed in “Discussion” section, the operative finding is not a measured rise toward the limit but a *monitoring blind spot*: the 0.100 mg L$^{-1}$ reporting limit that emerged in 2012 lies above the entire chronic ecotoxicological range for sensitive freshwater species (10–$50~\upmu \text {g/L}$) and at 56% of the CONAMA limit, so that 88% of post-2012 Zn measurements are censored across precisely the concentration band where diffuse-source (tyre and road wear) impacts on aquatic life would first become detectable.

### Spatial source factor contributions per UGRHI

**Fig. 9:**
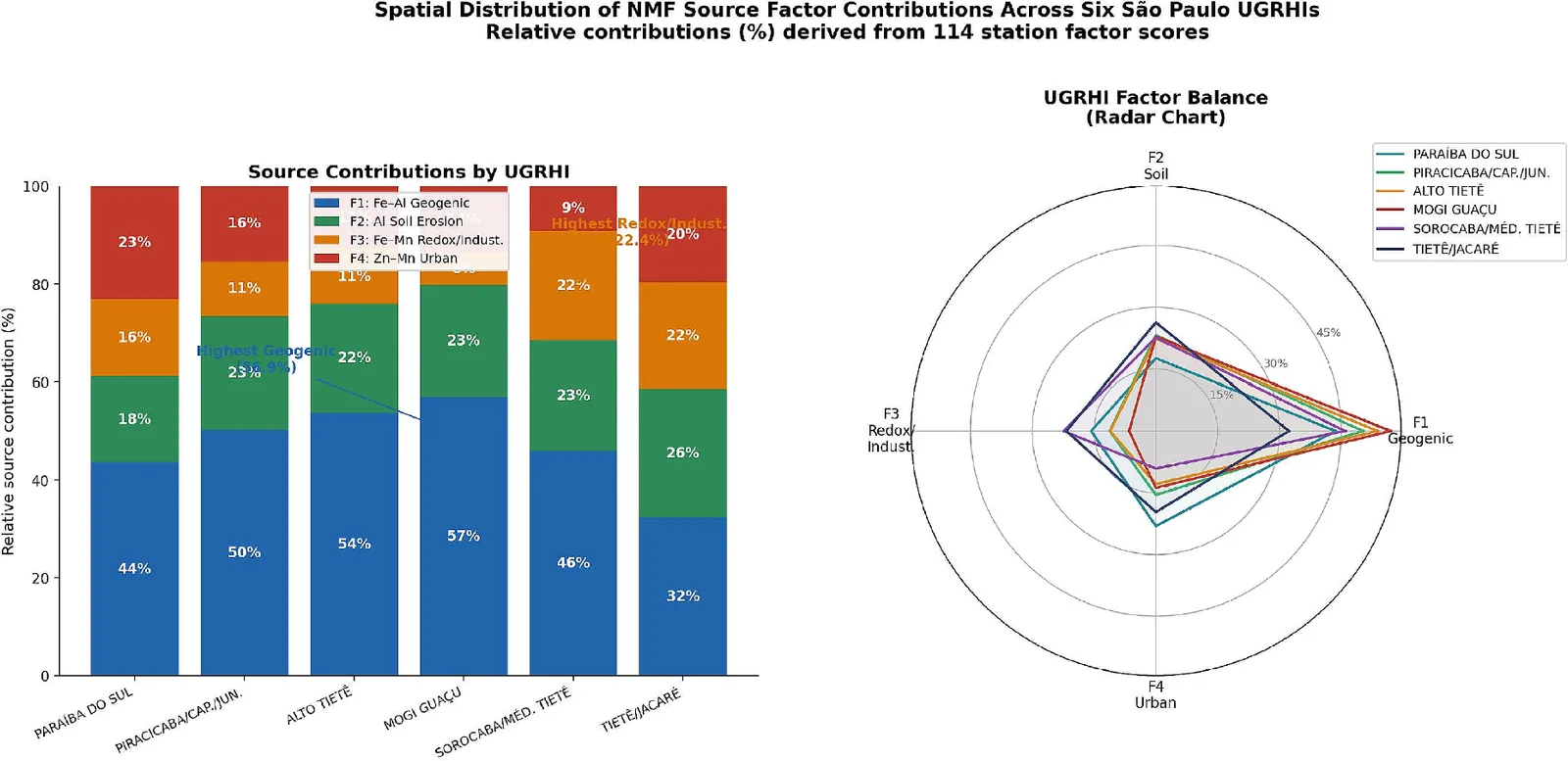
Spatial distribution of NMF source factor contributions across the six UGRHIs. Pie charts proportional to relative factor contributions (Table 11). Colour scheme: blue = F1 geogenic, green = F2 soil erosion, orange = F3 redox/industrial, red = F4 urban diffuse

Table 11 presents the relative NMF source factor contributions per UGRHI, normalised to unity. Figure 9 maps these contributions spatially.

**Table 11:** Relative NMF source-factor contributions per UGRHI (%), from the deterministic (NNDSVD) receptor solution

UGRHI	F1 geogenic (%)	F2 soil erosion (%)	F3 Redox/Indust. (%)	F4 urban (%)
02 – Paraíba do Sul	43.6	17.6	15.7	**23**.**1**
05 – Piracicaba/Capivari/Jundiaí	50.2	23.2	11.2	15.5
06 – Alto Tietê	53.7	22.3	11.1	12.9
09 – Mogi Guaçu	**56**.**9**	22.9	6.5	13.8
10 – Sorocaba/Médio Tietê	45.9	22.6	**22**.**4**	9.1
13 – Tietê/Jacaré	32.3	**26**.**3**	21.7	19.7

F1 = Fe–Al geogenic; F2 = Al soil erosion; F3 = Mn redox/industrial; F4 = Zn-bearing urban diffuse. Highest value per factor in bold. Reproducible from the deposited pipeline

**Mogi Guaçu** (F1 = 56.9%) exhibits the highest geogenic factor contribution of any UGRHI. Its Fe violation rate (96.1%) is the highest in the dataset, yet the receptor model assigns this predominantly to Factor 1 (geogenic weathering), not to the industrial factor (Factor 3 = 6.5%), and its industrial-metal violations are among the lowest recorded (Cu 23.7%, Mn 23.4%). This strongly implies that Mogi Guaçu’s Fe violations are a regulatory artefact of the CONAMA 357/2005 standard, which does not account for the naturally elevated Fe background of this predominantly Latosol catchment.

**The Tietê corridor** concentrates the anthropogenic metal burden. Sorocaba/Médio Tietê (UGRHI 10) records the highest industrial/redox factor contribution (F3 = 22.4%) together with the highest manganese violation rate in the dataset (84.7%) and elevated copper (58.2%) and nickel (22.6%). Immediately upstream, Alto Tietê (UGRHI 06), draining the São Paulo metropolitan region, records the highest copper (71.7%), zinc (32.8%), and nickel (46.7%) violation rates of any UGRHI. The convergence of elevated copper, manganese, nickel, and zinc along the Tietê axis points to concentrated industrial discharge and urban runoff, consistent with the metallurgical, automotive, and paper/pulp sectors of the Sorocaba and metropolitan corridors.

**Tietê/Jacaré and Paraíba do Sul** show lower and more mixed contributions. The Paraíba do Sul basin, characterised separately with respect to station redundancy and optimisation (Tavares & de Souza Sampaio, 2026), exhibits a predominantly geogenic signature (Cu 14.6%, Ni 4.3% violation) overlain by moderate industrial and urban inputs from the historic Volta Redonda steelworks and the São José dos Campos–Taubaté–Resende corridor; the present receptor analysis complements that network-level perspective with source-specific metal attribution across its CETESB stations. Lead is deliberately excluded as a spatial discriminator: 81% of Pb records are below detection, and in 39% of those the reporting limit itself exceeds the CONAMA Pb standard (0.01 mg L$^{-1}$), rendering basin-level Pb violation rates uninterpretable (Group B).

### CONAMA compliance and geogenic misattribution

**Fig. 10:**
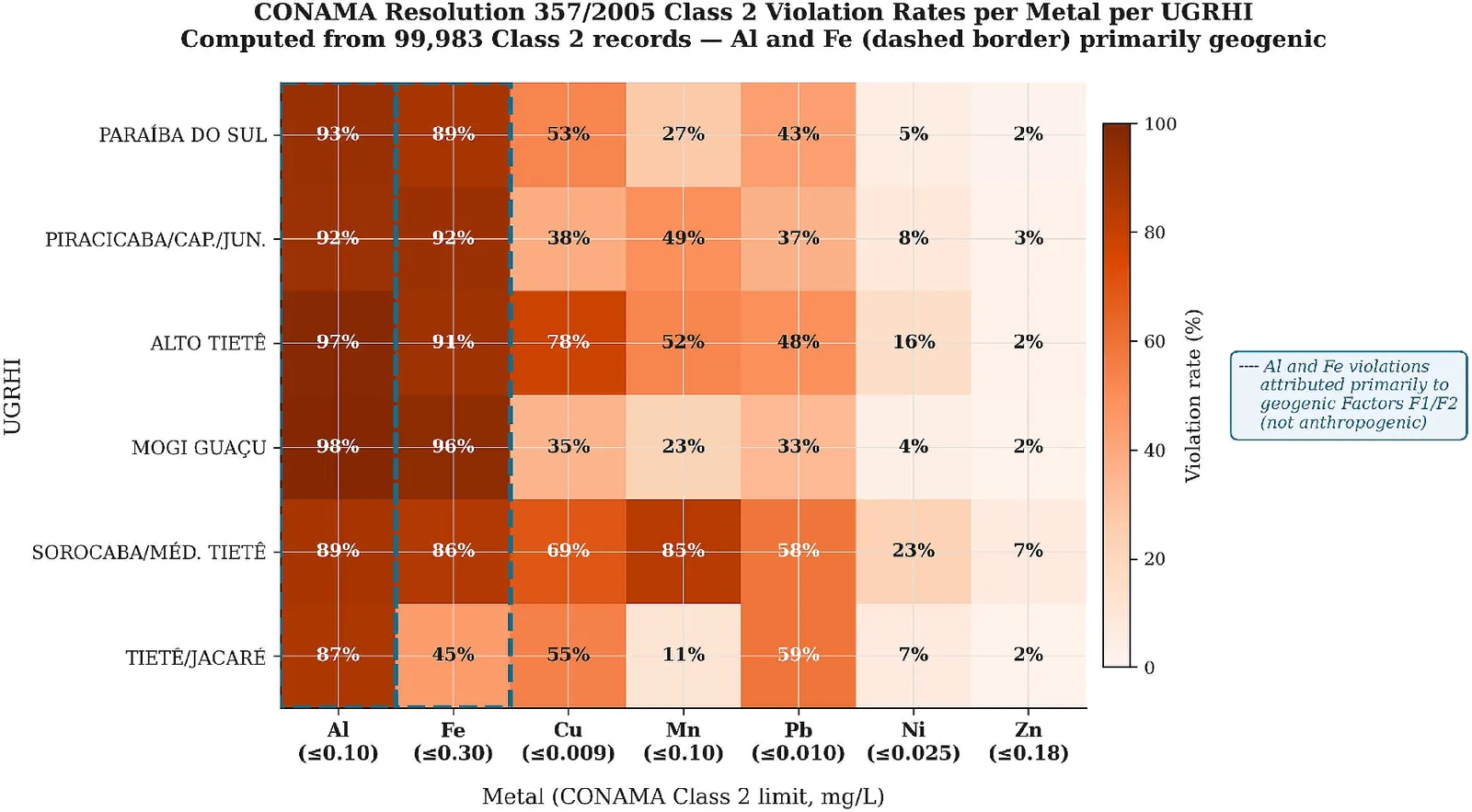
CONAMA Class 2 violation rates (%) per metal per UGRHI. Colour scale from white (0%) to deep red (>90%). Metals with primarily geogenic factor attribution (Al, Fe from Factors F1/F2) are highlighted with a dashed border. Rates here are computed on the CONAMA Class 2 subset of the record (the metal analyses from waters formally classified as Class 2, to which these limits apply), as are the basin-level rates cited in the text (e.g. Mogi Guaçu Fe 96.1%, Sorocaba Mn 84.7%); the system-wide rates reported in the Abstract and “Dataset characterisation and CONAMA compliance overview” section (Al 91.6%, Fe 88.2%) are computed across all monitored waters and are ≈1 percentage point lower

The violation heatmap (Fig. 10) and Table 12 reveal a clear two-tier structure. Metals attributed by the NMF model primarily to anthropogenic factors (Cu, Pb, Cd, Hg) show heterogeneous violation patterns with high spatial variability — reflecting genuine anthropogenic point-source concentration in specific UGRHIs. Metals attributed primarily to geogenic factors (Al, Fe) show uniformly high violation rates (>85%) across all UGRHIs regardless of industrial activity, which is the expected pattern for a geogenic baseline exceeding the regulatory standard.

**Table 12:** CONAMA Class 2 violation rates (%) per UGRHI for seven metals, computed as the fraction of all samples (1978–2026) exceeding the fixed CONAMA limit (below-detection records counted as non-exceeding)

UGRHI	*Al*	Cu	*Fe*	Mn	Pb	Zn	Ni
02 – Paraíba do Sul	*83.5*	14.6	*81.4*	24.5	2.9	1.6	4.3
05 – Piracicaba/Capivari/Jundiaí	*90.9*	29.8	*91.2*	49.8	6.8	2.8	8.3
06 – Alto Tietê	*98.6*	71.7	*92.5*	83.0	12.7	32.8	46.7
09 – Mogi Guaçu	*97.6*	23.7	*96.1*	23.4	6.0	2.1	4.0
10 – Sorocaba/Médio Tietê	*87.9*	58.2	*85.5*	84.7	7.4	7.2	22.6
13 – Tietê/Jacaré	*69.4*	30.4	*44.8*	11.0	6.3	2.2	7.0

Geogenic metals (Al, Fe) italicized. Lead is reported for completeness but is analytically uninterpretable (81% censored; reporting limit exceeds the CONAMA Pb standard in 39% of censored records). Reproducible from the deposited pipeline

The Mogi Guaçu anomaly is most diagnostic. Its Fe violation rate (96.1%) is the highest in the dataset, yet its Factor 3 (industrial) contribution is the lowest (6.5%), and its Factor 1 (geogenic) contribution is the highest (56.9%). This inverse relationship between industrial factor dominance and Fe violation rate is the strongest evidence of geogenic misattribution: in a basin with minimal industrial activity and maximum geogenic factor dominance, Fe violations are near-universal—a pattern that cannot be explained by anthropogenic loading and is entirely consistent with background Latosol Fe enrichment.

### Geochemical ratios as independent source evidence

To provide independent geochemical evidence for the NMF factor assignments, characteristic inter-element ratios were computed from per-UGRHI median concentrations (Table 13). These ratios serve as fingerprints for distinguishing geogenic from anthropogenic sources (Yang et al., 2023).

**Table 13:** Characteristic geochemical ratios per UGRHI (computed from per-UGRHI median concentrations, all years combined) and for all basins pooled

UGRHI	Fe/Al	Mn/Fe	Zn/Cu	Pb/Cd	Ni/Zn	Zn/Al
02—Paraíba do Sul	2.70	0.112	6.00	10.0	0.667	0.091
05—Piracicaba/Capivari/Jundiaí	2.02	0.050	12.00	10.0	0.333	0.061
06—Alto Tietê	1.73	0.081	6.00	12.5	0.167	0.084
09—Mogi Guaçu	2.00	0.050	8.00	10.0	0.500	0.040
10—Sorocaba/Médio Tietê	2.00	0.115	6.00	20.0	0.333	0.060
13—Tietê/Jacaré	1.25	0.200	1.00	20.0	1.000	0.050
All basins	2.13	0.057	5.56	10.0	0.400	0.061

Fe/Al and Mn/Fe are geogenic discriminators; Zn/Cu, Pb/Cd, and Ni/Zn are anthropogenic source fingerprints; Zn/Al indicates urban diffuse loading relative to geogenic background

The Fe/Al ratio (mean 2.13 across all basins) is consistent with values reported for lateritic Latosol weathering profiles in tropical Brazil, where Fe$_2$O$_3$/Al$_2$O$_3$ mass ratios of 0.5–3.0 are characteristic of the pedological landscape (Zahan et al., 2023). This corroborates the NMF assignment of Al and Fe to geogenic Factors F1 and F2, independently of the multivariate model. The Mn/Fe ratio (mean 0.057) is low, consistent with geogenic co-mobilisation under moderately oxic conditions rather than strongly reducing environments, and argues against the dominance of anoxic redox-driven Mn dissolution in most basins; Mogi Guaçu (0.050) and Piracicaba (0.050) have the lowest Mn/Fe, consistent with their predominantly geogenic factor profile (F1 dominant). Sorocaba (Mn/Fe = 0.115) and Tietê/Jacaré (0.200) have elevated Mn/Fe ratios, consistent with additional Mn input from industrial or redox sources (Factor F3 dominant in Sorocaba; F4 dominant in Tietê/Jacaré).

The Pb/Cd ratio (10–20 across UGRHIs) falls within the range characteristic of metallurgical and industrial co-emission sources globally (Yang et al., 2023), independently corroborating the NMF industrial factor (F3) assignment and the strong Cd–Pb Pearson correlation (r=+0.792). The Ni/Zn ratio is highest in Tietê/Jacaré (1.00), consistent with combined contributions from tyre wear (Zn-dominant) and infrastructure corrosion (Ni-associated), as documented for urban catchments globally (Soetan et al., 2025).

### Temporal evolution of NMF source factors

Table S2 (Supplementary Material) reports the dominant normalised metal loadings for the temporally dynamic source factors (F3 and F4) across the two regulatory sub-periods. The temporal analysis reveals not merely a quantitative shift in source contributions, but a qualitative change in factor composition that directly confirms the article’s central narrative.

Because the sub-period solutions are subject to the same rotational ambiguity discussed for the full-period model (“Limitations and future directions” section), we interpret them at the level of dominant-metal identities and the direction of change between periods rather than through precise loading values; the full normalised loadings are given in Table S2.

In the pre-CONAMA period (1990–2005), lead carried a non-negligible loading within the anthropogenic-associated factors, appearing at up to 0.15 in a mixed barium–lead factor (F4: Ba =0.41, Al =0.19, Pb =0.15)—a fingerprint consistent with the co-emission of Pb (from leaded-fuel combustion, battery manufacturing, and smelting) characteristic of Brazil’s pre-2005 industrial baseline. Critically, no factor in this period is zinc-dominated (the maximum Zn loading is only 0.11), indicating that a distinct urban diffuse zinc source had not yet emerged.

In the post-CONAMA period (2006–2026), the lead signature collapses: Pb falls to ≤0.03 across all four factors, consistent with the documented decline in Pb following the leaded-fuel phase-out and CONAMA 357/2005 enforcement. Simultaneously, a distinct zinc-led urban diffuse factor emerges and strengthens (F4: Zn =0.49, Mn =0.29)—the urban source signature identified in the full-period model. The geogenic Fe–Al factor and the Fe-dominated redox/industrial factor (F3: Fe =0.77→0.63) persist across both periods, confirming that the source structure is temporally stable except for precisely the two changes that track regulation directly.

These temporal NMF results confirm that the full-period model captures a meaningful average of two distinct source regimes rather than an artefact of collapsing temporal dynamics. The regulatory narrative (Pb-associated industrial sources declined; Zn-dominated urban diffuse sources grew) is reproduced independently in both sub-period models, strengthening the robustness of the main conclusions.

## Discussion

### The geogenic regulatory artefact: implications for CONAMA 357/2005

The receptor modelling results establish that the overwhelming majority of CONAMA Class 2 aluminium and iron violations in São Paulo’s river basins are attributable to geogenic Factor 1 (Fe–Al weathering) and Factor 2 (Al soil erosion), not to anthropogenic contamination. This finding has a direct regulatory corollary. If enforcement of Al and Fe standards in geogenically elevated catchments (e.g., Mogi Guaçu) is predicated on the assumption that measured concentrations are anthropogenic, regulatory resources are being directed toward non-remediable targets while genuinely anthropogenic sources of other metals receive comparatively less scrutiny.

This regulatory science contribution parallels the evolution of metal quality standards in other jurisdictions. The US EPA’s Clean Water Act Section 304(a) has progressively adopted a “reference condition” methodology that calibrates ambient water quality criteria to natural background concentrations in comparable geologies, distinguishing anthropogenic additions from natural baseline. The WFD Article 4 and associated technical guidance require member states to derive “natural background concentrations” for naturally enriched metals and to apply standards only to anthropogenically elevated fractions (European Parliament and Council, 2000). Brazil’s CONAMA 357/2005 lacks an equivalent mechanism, having adopted WHO drinking water guidelines calibrated to temperate-zone geochemistry. Establishing geology-specific reference conditions for Al and Fe in São Paulo’s Latosol-dominated catchments—using the receptor modelling evidence presented here as the geogenic contribution baseline—would constitute a significant regulatory advance with direct applicability to other tropical Brazilian states with similar pedological profiles.

### Zinc: a monitoring blind spot for a diffuse-source metal

Zinc’s behaviour represents the most consequential finding for future water quality management in the study area, but not in the form the median trend first suggests. Since the apparent +362% increase is a reporting-limit artefact and the true exceedance fraction has declined (“Censoring-robust trend verification” section), the consequential finding is instead structural: the current 0.100 mg L$^{-1}$ reporting limit renders the monitoring network blind to zinc across precisely the concentration band where diffuse-source impacts on aquatic ecosystems would first emerge. Zinc’s exclusive attribution to the urban diffuse Factor 4, its co-occurrence with Mn (a known tyre rubber accelerant), and the positive Ni–Zn correlation still provide circumstantial evidence that the urban diffuse source is growing in relative importance, consistent with the global mechanistic literature on tyre and road wear particles (Baensch-Baltruschat et al., 2020); what the current monitoring cannot do is quantify that source against ecologically relevant thresholds.

The health and ecological relevance of this trend is well-established at the global scale. Tian et al. (2021) demonstrated that TRWP-derived Zn in urban stormwater is the primary acute toxicant causing mass mortality of coho salmon (*Oncorhynchus kisutch*) in US Pacific Northwest streams; the responsible chemical—N-(1,3-dimethylbutyl)-N’-phenyl-p-phenylenediamine (6PPD-quinone)—is co-transported with Zn in TRWP, and its aquatic toxicity threshold ($< 0.1~\upmu \text {g/L}$ for salmon) is orders of magnitude below Zn regulatory limits. Analogous spatiotemporal assessments of metal pollution and human health risks in urban river systems have similarly identified Zn as a priority pollutant of diffuse origin (Soetan et al., 2025). The ecological risk assessment framework applied to Zn in sediments of similar tropical river systems (South and Southeast Asia) consistently identifies Zn as a moderate-to-high ecological risk driver at concentrations approaching regulatory limits (Cao et al., 2026).

For human ingestion specifically, zinc is of comparatively low concern: the WHO drinking-water guideline (3 mg L$^{-1}$) is roughly seventeen times the CONAMA Class 2 aquatic standard, and the CONAMA standard is protective of aquatic ecosystems rather than human ingestion. The ecological pathway is where the sensitivity lies. Aquatic toxicity thresholds for freshwater invertebrates and fish are considerably lower: chronic effect concentrations for sensitive species range from 10–$50~\upmu \text {g/L}$ in soft to moderately hard water (Aura et al., 2024). This is the crux of the monitoring blind spot: the 0.100 mg L$^{-1}$ ($100~\upmu \text {g/L}$) reporting limit that has governed the CETESB Zn record since 2012 lies entirely above this chronic-toxicity range and at 56% of the CONAMA limit, so that the 88% of post-2012 Zn measurements returned as “below detection” span the whole band (10–$100~\upmu \text {g/L}$) in which chronic ecological harm to sensitive species would occur. The network can therefore confirm neither compliance nor exceedance of ecologically protective concentrations: a river at a genuinely harmful $40~\upmu \text {g/L}$ and one at a benign $5~\upmu \text {g/L}$ are recorded identically as “<0.100”. Whether or not zinc is presently harming aquatic life in these basins is, on the current monitoring design, unknowable—and that unknowability is itself the governance finding.

The governance gap is therefore twofold. First, it is analytical: a monitoring programme cannot manage what it cannot measure, and the priority recommendation emerging from this study is that CETESB (and analogous networks) lower the Zn reporting limit to at least $10~\upmu \text {g/L}$ so that the ecologically relevant range becomes observable, enabling a genuine future trend assessment that the present record cannot support. Second, it is regulatory: CONAMA 357/2005 addresses Zn through a point-source effluent framework, but TRWP-derived Zn enters waterways as diffuse stormwater input, and no Brazilian regulatory framework currently restricts TRWP generation, mandates stormwater Zn monitoring, or requires treatment of urban road runoff before discharge. The receptor modelling and censoring-robust evidence presented here provide the quantitative basis for both the detection-limit reform and the inclusion of Zn/TRWP in Brazil’s next revision of the National Water Resources Plan.

### Nickel as a forward-looking governance indicator

Nickel’s median trend is superficially increasing (τ=+0.461, p=0.006), but it fails the same censoring-robust test as zinc, and more severely: the detected-only median shows no significant trend (τ=+0.071, p=0.58) and the exceedance fraction declines (τ=-0.600, p<0.001; Table 10), against a background of 88% below-detection at a reporting limit (0.020 mg L$^{-1}$) that sits at the median, so that the rise in censoring from 80% to 97% over the period mechanically inflates the median-based trend (Table 2). We therefore make no claim of a present nickel increase. Nickel’s interest is instead forward-looking and precautionary in the context of Brazil’s energy and transport transition. The reported median (0.020 mg L$^{-1}$) sits just below the CONAMA Class 2 limit (0.025 mg L$^{-1}$) and below WHO drinking water guidelines (0.07 mg L$^{-1}$); chronic aquatic toxicity thresholds for Ni range from 8–$150~\upmu \text {g/L}$ depending on hardness and species sensitivity (Yang et al., 2023), so the ecologically relevant range again straddles the reporting limit and would be poorly resolved by the current monitoring design. Nickel-rich NMC and NCA lithium-ion battery cathodes contain 40–80% Ni by mass, and end-of-life vehicle battery recycling and manufacturing waste from the expanding Brazilian EV supply chain represent plausible future point sources not yet on CETESB’s parameter priority list. The value of the present record is thus as a *pre-transition baseline*: it establishes that, as of the mid-2020s, nickel exceedance of the CONAMA limit is stable-to-declining, providing a defensible reference against which genuine future increases—should they occur as EV penetration rises under Brazil’s Mover programme (30% electrified vehicle sales targeted by 2030)—can be detected, but only if the reporting limit is lowered into the ecotoxicologically relevant range.

### The Tietê Corridor as the multi-domain impairment hotspot

The Tietê corridor’s dominance across multiple contamination indicators is consistent with the convergence of concentrated metallurgical, automotive-parts, and paper/pulp industries with dense urban runoff. Sorocaba/Médio Tietê (UGRHI 10) carries the highest industrial/redox factor contribution (Factor 3 = 22.4%) and the highest manganese violation rate in the dataset (84.7%), while the adjacent Alto Tietê (UGRHI 06), draining the São Paulo metropolitan region, records the highest copper (71.7%), zinc (32.8%), and nickel (46.7%) violation rates. This convergence of elevated anthropogenic metals along the Tietê axis is consistent with independently documented chronic non-compliance of the same basins under conventional water-quality parameters, where Alto Tietê and Sorocaba/Médio Tietê record the highest dissolved-oxygen, BOD, and total-phosphorus violation rates of the CETESB network (Tavares & de Souza Sampaio, 2026). This finding mirrors patterns reported for heavily industrialised river sub-basins globally, where receptor modelling consistently identifies Fe–Mn redox and industrial factors as the dominant contamination drivers in zones of dense manufacturing activity (Silva et al., 2026; Yang et al., 2023). The convergence of the highest Factor 3 contribution with extreme multi-metal violation rates along the Tietê axis provides the quantitative basis for basin-specific enforcement prioritisation in São Paulo’s next UGRHI management plan review, a level of spatial specificity that aggregate compliance reporting cannot deliver.

### Limitations and future directions

Several limitations should be acknowledged. First, the NMF receptor model employed here does not provide bootstrap uncertainty quantification equivalent to EPA PMF 5.0’s rotational ambiguity analysis. The four-factor solution should be validated using EPA PMF with full uncertainty diagnostics (Q/Q$_\textrm{exp}$ ratio, displacement analysis—DISP, and bootstrap-enhanced DISP—BS-DISP) in a follow-up study. While NMF and EPA PMF share the non-negativity constraint and have been shown to produce consistent source profiles in comparative studies (Li et al., 2022), the rotational ambiguity inherent in non-negative factorisation can only be formally assessed through EPA PMF’s dedicated uncertainty protocols; the present NMF solution should therefore be regarded as exploratory source profile resolution pending full EPA PMF 5.0 validation. Second, the input matrix is constructed from station medians across the full 47-year period, collapsing temporal dynamics into a single spatial snapshot for the receptor model; separate models for defined regulatory periods (pre-/post-CONAMA 357, pre-/post-drought) would allow temporal resolution of source-factor evolution. Third, As data coverage is substantially lower than other metals ($n = 2{,}721$, limited to 2009–2025 at 75 stations), and the apparent Mann–Kendall trend (τ=+0.569, p=0.006) is an artefact of analytical censoring: 97.5% of As records carry a below-detection flag, and annual medians transition from values near the instrument detection limit (0.002 mg L$^{-1}$, 2009–2011) to the CONAMA Class 2 limit (0.010 mg L$^{-1}$, 2012–2025), which represents a change in detection-limit reporting practice rather than a genuine increase in As river concentrations. As is therefore classified in Group B alongside Hg. Fourth, mercury (Hg) presents a particular interpretative challenge: 86.4% of Hg records in the CETESB QUALAR database carry a below-detection flag, with reported values representing MDL substitutions (0.0001 or 0.0002 mg L$^{-1}$). Hg’s NMF factor loadings are consequently near zero across all four factors and should not be used to make source attribution inferences for this metal; Hg trends and violation rates are reported for completeness but are subject to the analytical censoring caveat. Fifth, and generalising the Hg and As cases, median-based trends for any heavily censored metal whose reporting limit changed over time are potentially confounded by that reporting change; this is why all trend interpretation here rests on the censoring-invariant exceedance analysis (“Censoring-robust trend verification” section) rather than on median trends alone. Complementary maximum-likelihood or Kaplan–Meier treatment of the censored distributions is recommended as further confirmation in follow-up work.

Finally, a limitation intrinsic to the secondary-data design of this study must be stated explicitly. Because the analysis draws on validated results distributed through the public CETESB QUALAR portal rather than on primary laboratory campaigns, sample-level quality-assurance records—certified-reference-material recoveries, procedural and field blank levels, per-batch spike recoveries, and individual filtration and container-preparation logs—are not retrievable and therefore cannot be reported here, notwithstanding the laboratory’s institutional ISO/IEC 17025 accreditation. We have mitigated this constraint in two ways: by documenting the per-metal analytical technique and the range of reported method detection limits over the study period (Table 2), and by providing three independent geochemical self-consistency tests (“Geochemical cross-validation of data integrity” section) that establish the internal integrity of the data—a documented censoring-robust regulatory decline, the expected redox-driven Mn seasonal signal, and the expected covariation of the geogenic metals with turbidity—without relying on any documentary QA record. These empirical checks constitute the strongest data-quality evidence available for a network-scale secondary dataset, but they are not a substitute for sample-level QA/QC metadata, and conclusions that would require such metadata (for example, absolute-concentration comparisons at the sub-microgram-per-litre level) are correspondingly avoided throughout.

## Conclusions

This study applies NMF receptor modelling to one of the largest multi-metal river monitoring datasets in Latin America—126,140 records of eleven trace metals at 114 stations across six São Paulo UGRHIs (1978–2026)—and reaches four principal conclusions:

1. Four geochemically interpretable source factors are resolved: (F1) Fe–Al geogenic weathering, (F2) Al soil erosion, (F3) Fe–Mn redox/industrial effluent, and (F4) Zn–Mn urban diffuse contamination. The Cd–Pb correlation (r=+0.792) provides independent corroboration of the industrial source factor, while Al–Fe (r=+0.786) confirms the geogenic weathering factor.
2. The 91.6% aluminium violation rate and 88.2% iron violation rate under CONAMA Class 2 are attributable primarily to geogenic Factors F1 and F2, not to anthropogenic contamination. This constitutes a geogenic regulatory artefact—the CONAMA 357/2005 limits for Al and Fe are calibrated to temperate-zone geochemistry and do not account for the naturally elevated Al$_2$O$_3$ and Fe$_2$O$_3$ contents of Brazilian Latosol pedologies. Adoption of a reference-condition methodology analogous to those in the US EPA and EU WFD frameworks is recommended.
3. Zinc shows the strongest *apparent* increasing median trend (τ=+0.727), but a censoring-invariant exceedance analysis shows this to be an artefact of a 2012 rise in the analytical reporting limit (0.020 to 0.100 mg L$^{-1}$): the fraction of samples truly exceeding the CONAMA limit in fact declines (τ=-0.477, p<0.001), and no metal shows a rising exceedance of its regulatory limit. The operative zinc finding is instead a *monitoring blind spot*—the 0.100 mg L$^{-1}$ reporting limit exceeds the chronic ecotoxicological range for sensitive freshwater species (10–$50~\upmu \text {g/L}$), rendering diffuse-source (tyre and road wear) zinc unmeasurable at ecologically relevant concentrations. Lowering the Zn and Ni reporting limits into that range, and extending CONAMA’s point-source architecture to diffuse stormwater inputs, are the priority recommendations.
4. Spatial source apportionment identifies the Tietê corridor (Alto and Médio Tietê) as the multi-metal impairment axis—with the highest copper (up to 71.7%), manganese (up to 84.7%), nickel (up to 46.7%), and zinc (32.8%) violation rates in the dataset—and Mogi Guaçu as the basin where iron violations (96.1%, the dataset maximum) are most clearly misattributed to non-existent anthropogenic sources, its industrial-metal violations being among the lowest recorded. This spatial contrast—near-universal geogenic Fe exceedance decoupled from anthropogenic-metal exceedance—is the strongest single line of evidence for the geogenic-misattribution thesis.

These findings position São Paulo’s metal contamination governance within the global pollution-transition pattern: measurable regulatory success for industrial point sources, alongside a detection-limit blind spot that prevents the monitoring network from confirming or refuting an emerging diffuse-source problem. Beyond the regional conclusions, the study carries a transferable methodological caution: in long-term monitoring records where reporting limits evolve, trend analyses based on median or MDL-substituted values can manufacture spurious “emerging-contaminant” signals, and censoring-invariant statistics such as fixed-threshold exceedance fractions are necessary to distinguish genuine environmental change from analytical reporting change. The specific demonstration that geogenic metal loads generate regulatory artefacts in tropical Latosol geologies is transferable to other rapidly industrialising regions with similar pedological profiles, including the Brazilian Amazon basin, Central Africa, and South and Southeast Asia.

## Supplementary Information

Below is the link to the electronic supplementary material.

Supplementary file1 (PDF 328 KB)

## Data Availability

The CETESB monitoring dataset is publicly available via the CETESB QUALAR platform (https://qualar.cetesb.sp.gov.br/). Processed data files and analysis scripts (Python 3.12) used to generate all figures and statistical results reported in this study are available from the corresponding author upon reasonable request. This study is part of a doctoral research programme at DEAMB/UERJ.
