# Supplementary material for: Source apportionment of eleven trace metals across six São Paulo river basins (1978–2026): receptor modelling identifies geogenic misregulation and a monitoring blind spot for diffuse-source zinc
Source: Environ Geochem Health. 2026 Aug 18;48(13):536. doi: 10.1007/s10653-026-03379-2 (PMC13486052; doi:10.1007/s10653-026-03379-2)
Supplement: Supplementary file 1 [file 10653_2026_3379_MOESM1_ESM.pdf]

Supplementary Material

Figure S1 (Supplementary Material, Fig. 11) presents the long-term concentration trajectories (annual medians, 2000–2025) for six representative metals spanning all three trajectory groups identified in the Mann-Kendall analysis: Zn and Ni (Group C, increasing); Cu (Group A, regulated declining); Hg (Group B, censored — 86.4% of records below analytical detection limit); Fe (Group A, partly geogenic); and Pb (Group B, statistically inconclusive). CONAMA Class 2 reference limits are indicated for each panel.

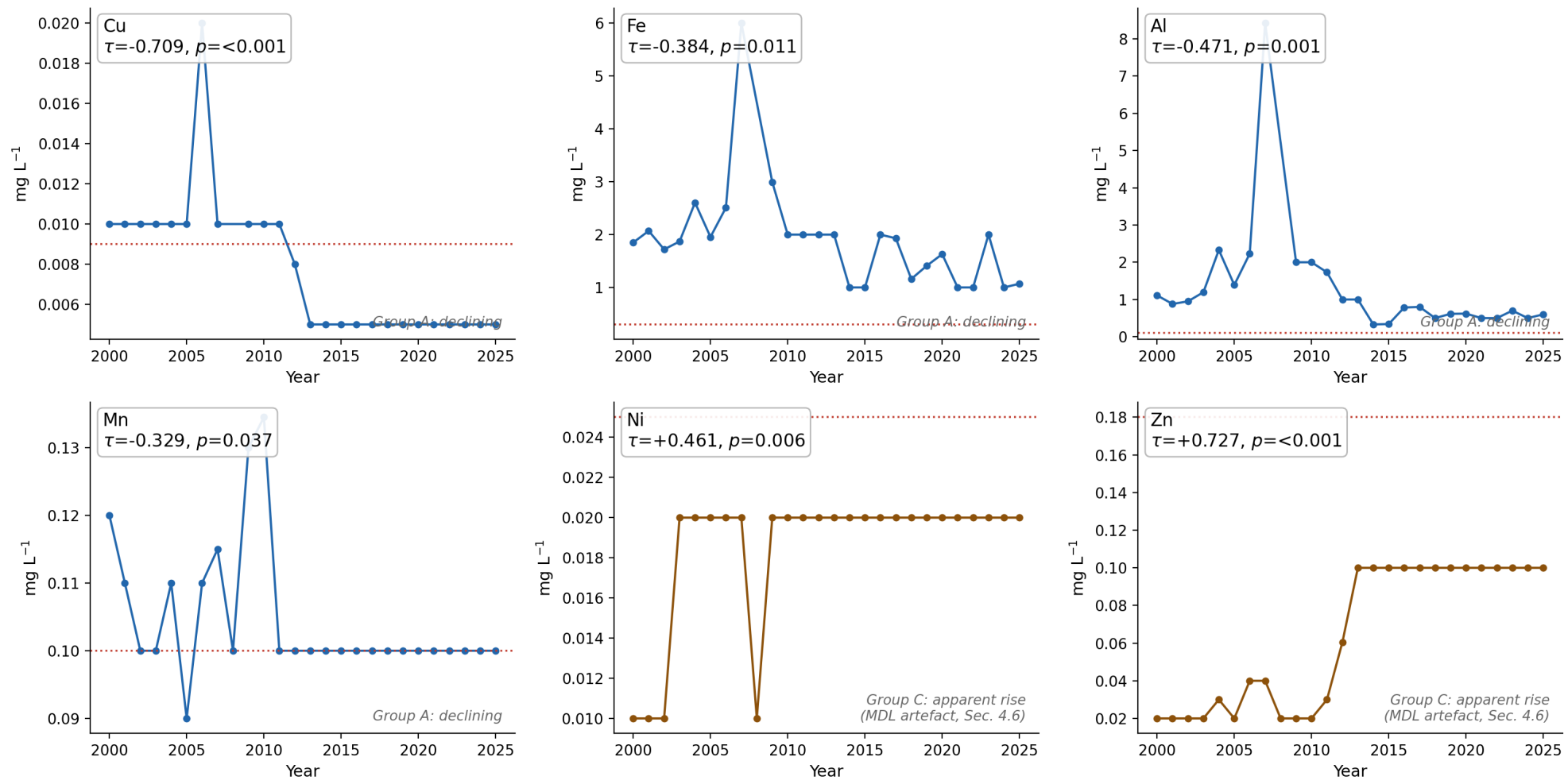

Fig. 11 Supplementary Figure S1. Annual median concentrations (2000–2025) for six representative trace metals (Cu, Fe, Al, Mn — Group A, declining; Ni, Zn — Group C, apparent median increase) across all six São Paulo UGRHIs combined. Each panel shows the Mann-Kendall τ statistic and p-value (top-left inset) and the CONAMA Resolution 357/2005 Class 2 reference limit (red dotted horizontal line). The Group C panels (Ni, Zn) are annotated to indicate that their positive median trends are detection-limit artefacts, as established by the censoring-robust analysis (Section 4.6). Source: CETESB QUALAR monitoring database (126,140 metal records, 114 stations, 1978–2026).

Table 14 Sub-period NMF factor loadings (normalised, deterministic NNDSVD), fitted separately to the pre- and post-CONAMA 357/2005 periods. The industrial Pb signature weakens and a zinc-led urban diffuse factor strengthens post-2006. Reproducible from the deposited pipeline.

Period	Factor	Al	Fe	Mn	Cu	Zn	Ni	Ba	Pb	Cd	Hg	As
Pre-2006 (n=56)	F1	0.40	0.57	0.00	0.00	0.00	0.00	0.01	0.02	0.00	0.00	0.00
	F2	0.68	0.00	0.16	0.03	0.11	0.02	0.00	0.00	0.00	0.00	0.00
	F3	0.00	0.77	0.15	0.00	0.00	0.00	0.05	0.03	0.00	0.00	0.01
	F4	0.19	0.00	0.11	0.01	0.00	0.06	0.41	0.15	0.02	0.00	0.05
Post-2006 (n=107)	F1	0.20	0.77	0.00	0.00	0.00	0.00	0.01	0.00	0.00	0.00	0.00
	F2	0.95	0.00	0.01	0.00	0.01	0.00	0.02	0.00	0.00	0.00	0.00
	F3	0.00	0.63	0.26	0.02	0.00	0.01	0.07	0.01	0.00	0.00	0.00
	F4	0.00	0.00	0.29	0.01	0.49	0.06	0.08	0.03	0.00	0.00	0.02
